# People Living with Chronic Pain Experience a High Prevalence of Decision Regret in Canada: A Pan-Canadian Online Survey

**DOI:** 10.1177/0272989X251326069

**Published:** 2025-03-22

**Authors:** Florian Naye, Yannick Tousignant-Laflamme, Maxime Sasseville, Chloé Cachinho, Thomas Gérard, Karine Toupin-April, Olivia Dubois, Jean-Sébastien Paquette, Annie LeBlanc, Isabelle Gaboury, Marie-Ève Poitras, Linda C. Li, Alison M. Hoens, Marie-Dominique Poirier, France Légaré, Simon Décary

**Affiliations:** School of Rehabilitation, Université de Sherbrooke, Faculty of Medicine and Health Sciences, Sherbrooke, QC, Canada; School of Rehabilitation, Université de Sherbrooke, Faculty of Medicine and Health Sciences, Sherbrooke, QC, Canada; VITAM Research Center for Sustainable Health, Quebec Integrated University Health and Social Services Center (CIUSSS de la Capitale-Nationale), QC, Canada; Université Laval, Faculty of Nursing, QC, Canada; School of Rehabilitation, Université de Sherbrooke, Faculty of Medicine and Health Sciences, Sherbrooke, QC, Canada; Patient-research partner; School of Rehabilitation, Université de Sherbrooke, Faculty of Medicine and Health Sciences, Sherbrooke, QC, Canada; School of Rehabilitation Sciences, University of Ottawa, Faculty of Health Sciences, Ottawa, ON, Canada; Department of Pediatrics, University of Ottawa, Faculty of Medicine, Ottawa, ON, Canada; Institut du Savoir Montfort, Ottawa, ON, Canada; School of Rehabilitation, Université de Sherbrooke, Faculty of Medicine and Health Sciences, Sherbrooke, QC, Canada; VITAM Research Center for Sustainable Health, Quebec Integrated University Health and Social Services Center (CIUSSS de la Capitale-Nationale), QC, Canada; Department of Family and Emergency Medicine, Université Laval, Faculty of Medicine, QC, Canada; VITAM Research Center for Sustainable Health, Quebec Integrated University Health and Social Services Center (CIUSSS de la Capitale-Nationale), QC, Canada; Department of Family and Emergency Medicine, Université Laval, Faculty of Medicine, QC, Canada; Université de Sherbrooke, Faculty of Medicine and Health Sciences, Department of Family Medicine and Emergency Medicine, Sherbrooke, QC, Canada; Université de Sherbrooke, Faculty of Medicine and Health Sciences, Department of Family Medicine and Emergency Medicine, Sherbrooke, QC, Canada; Department of Physical Therapy, University of British Columbia, Vancouver, BC, Canada; Arthritis Research Canada, Vancouver, BC, Canada; Patient-research partner; Department of Physical Therapy, University of British Columbia, Vancouver, BC, Canada; Arthritis Research Canada, Vancouver, BC, Canada; Patient-research partner; VITAM Research Center for Sustainable Health, Quebec Integrated University Health and Social Services Center (CIUSSS de la Capitale-Nationale), QC, Canada; Department of Family and Emergency Medicine, Université Laval, Faculty of Medicine, QC, Canada; Canada Research Chair in Shared Decision Making and Knowledge Translation, Université Laval, Quebec City, QC, Canada; School of Rehabilitation, Université de Sherbrooke, Faculty of Medicine and Health Sciences, Sherbrooke, QC, Canada

**Keywords:** decision regret scale, decision regret, chronic pain, survey

## Abstract

**Background:**

(1) To estimate the prevalence of decision regret in chronic pain care, and (2) to identify factors associated with decision regret.

**Design:**

We conducted a pan-Canadian cross-sectional online survey and reported the results following the Checklist for Reporting of Survey Studies guidelines. We recruited a sample of adults experiencing chronic noncancer pain. We used a stratified proportional random sampling based on the population and chronic pain prevalence of each province. We measured decision regret with the Decision Regret Scale (DRS) and decisional needs with the Ottawa Decision Support Framework. We performed descriptive analysis to estimate the prevalence and level of decision regret and multilevel multivariable regression analysis to identify factors associated with regret according to the STRengthening Analytical Thinking for Observational Studies recommendations.

**Results:**

We surveyed 1,649 people living with chronic pain, and 1,373 reported a most difficult decision from the 10 prespecified ones, enabling the collection of a DRS score. On a scale ranging from 0 to 100 where 1 reflects the presence of decision regret and 25 constitutes important decision regret, the mean DRS score in our sample was 28.8 (*s* = 19.6). Eighty-four percent of respondents experienced some decision regret and 50% at an important level. We identified 15 factors associated with decision regret, including 4 personal and 9 decision-making characteristics, and 2 consequences of the chosen option. Respondents with low education level and higher decisional conflict experienced more decision regret when the decision was deemed difficult.

**Conclusions:**

This pan-Canadian survey highlighted a high prevalence and level of decision regret associated with difficult decisions for pain care. Decision making in pain care could be enhanced by addressing factors that contribute to decision regret.

**Highlights:**

- We conducted an online pan-Canadian survey and collected responses from a wide diversity of people living with chronic pain.
- More than 84% of respondents experienced decision regret and approximately 50% at an important level.
- We identified 15 factors associated with decision regret, including 4 personal and 9 decision-making characteristics, and 2 consequences of the chosen option.
- Our pan-Canadian survey reveals an urgent need of a shared decision-making approach in chronic pain care that can be potentiated by targeting multiple factors associated with decision regret.

## Introduction

People living with chronic pain face multiple difficult decisions that require addressing their specific decisional needs.^1,2^ Decisional needs are the support and information that people need to make a decision.^3^ If these decisional needs are met, the patient–clinician dyad is more likely to choose the best option for the patient’s condition.^1^ If these needs are unmet, decisional conflict can arise and lead to decision regret.^1^ In a recent survey across all Canadian provinces, we found that one-third of people living with chronic pain experienced clinically significant decisional conflict following a decision related to their condition and deemed difficult.^2^ In this context, measuring decision regret is valuable as a proxy measure to appraise the quality of the decision-making process.^4–7^ The prevalence of decision regret in difficult chronic pain decisions is still unknown.

Decision regret is “a feeling of distress or remorse following a decision,”^4^ and it arises when people estimate that they could have attained better results with another option.^8^ Decision regret is commonly measured in the research context with the Decision Regret Scale.^5,9^ Decision regret is associated with multiple negative consequences for the individuals including poorer physical and psychological health,^4,10,11^ increased pain,^12^ increased financial difficulty,^12^ and poorer well-being^13^ and quality of life.^4,10–12,14–16^ Decision regret also affects the experience with the health care system due to reduced satisfaction with the consultation.^4^ Reducing decision regret is necessary to enhance clinical outcomes and experiences of people living with chronic pain.

Shared decision making (SDM) can reduce decision regret. According to an updated Cochrane review, low-risk-of-bias studies on patient decision aids (i.e., one type of SDM interventions) shows effectiveness in decreasing decision regret.^17^ Among these studies, none specifically addressed chronic pain. As more than 1.6 billion individuals globally live with chronic pain^18–21^ and face multiple difficult decisions,^2^ there is a critical need to develop and implement SDM interventions. According to the International Patient Decision Aids Standards, the first step in developing a high-quality intervention is understanding the users’ needs.^22^ Identifying factors associated with decision regret is of great value to reach their specific decisional needs to enhance clinical practice and to inform the development of an intervention.

The aims of this study were 1) to estimate the prevalence and level of decision regret in chronic pain care and 2) to identify factors associated with decision regret.

## Methods

### Study Design and Settings

We conducted a pan-Canadian cross-sectional online survey, adhering to our previously published protocol.^23^ We reported data following the Checklist for Reporting of Survey Studies (CROSS) guidelines.^24^ We performed statistical analyses following the STRengthening Analytical Thinking for Observational Studies (STRATOS) recommendations.^25^

### Patient Involvement

Three patient–partners living with chronic pain were part of the steering committee having developed this protocol and were listed as coauthors. Two patient partners were also a rehabilitation professionals. Two patient partners had more than 5 y of experience in health research, and a novice patient partner was integrated in a capacity-building perspective. All the patient partners had training by the Strategy for Patient-Oriented Research SUPPORT units in Canada. The patient partners identified the tasks that were most meaningful for them. We discussed and decided appropriate compensation for their time.

### Respondents

We recruited a randomly selected sample of citizens, permanent residents, or refugees living in Canada, aged 18 y or older, who could read, write, and understand French or English and who were experiencing either primary chronic pain (e.g., low-back pain) or secondary chronic pain (e.g., pain from persistent inflammation)^26^ from the panel of Leger Marketing (https://leger360.com/). This private research analytical firm maintains a panel of 500,000 representative members of Canadian society with Internet access across the 10 Canadian provinces. The International Association for the Study of Pain defined chronic primary pain as pain in 1 or more anatomical regions that persists or recurs for longer than 3 mo and that cannot be better accounted for by another chronic pain condition^27^ and chronic secondary pain as chronic pain that is linked to other diseases as the underlying cause, for which pain may initially be regarded as a symptom.^26^ We excluded respondents with chronic cancer pain or chronic pain after cancer treatment.^28^ We excluded people living with chronic pain due to cancer because their decision-making context often involves higher-stakes decisions related to life expectancy, palliative care, and curative treatments, which differ fundamentally from the long-term symptom management focus in noncancer chronic pain. Cancer-related pain management also frequently includes intensive treatments, such as chemotherapy or radiation, shaping distinct decisional needs. The psychological impact of a life-threatening diagnosis may alter emotional and cognitive approaches to decision making, potentially leading to different experiences of regret.^29^ People living with cancer face complex treatment goals, balancing immediate pain relief with side effects and survival outcomes, which introduces confounding factors that differ from the goals of individuals with noncancer chronic pain.

We employed a stratified proportional random sampling: 1) the sample was stratified based on Canadian provinces (Alberta, British Columbia, Manitoba, Newfoundland and Labrador, New Brunswick, Nova Scotia, Ontario, Prince Edward Island, Quebec, and Saskatchewan), 2) the sample was adjusted proportionally according to both population size and the prevalence of chronic pain,^30,31^ and 3) we used Léger Marketing’s software to randomly select participants. After each solicitation, the next random sample was generated, accounting for the characteristics of previous participants (e.g., sex, age, ethnic and cultural backgrounds, education level) to ensure a more representative sample.

### Data Collection

The survey included a question on language preference (English or French) and 3 sections including the characteristics of participants. The full questionnaire contained 50 questions and took 30 min to complete. No standardized questionnaire was available to assess the decisional needs for people living with chronic primary and secondary pain. We developed a questionnaire with 6 domains: 1) difficult decisions, 2) health care needs, 3) decisional conflict, 4) decision regret, 5) decisional needs, and 6) characteristics of the participants.

#### Dependent variable: Decision regret

We used the Decision Regret Scale (DRS) to collect data on decision regret. It is composed of 5 items rated on a 5-level Likert scale (1 = *strongly agree*, 5 = *strongly disagree*).^4^ After transformation following the user manual of the instrument, a continuous score of decision regret is obtained (between 0 = *absence of decision regret* and 100 = *maximum decision regret*).^32^ There is no standardized cutoff for this measurement instrument, but DRS scores >0 and >25 are commonly used to reflect the presence of decision regret and the presence of an important level of decision regret, respectively.^33^ This scale shows satisfactory measurement properties for different kinds of health-related decisions.^4,34–36^ We used the French version in the context of another study in the French–Canadian population.^37,38^

#### Independent and adjustment variables

We describe the variables and data collection methods in Table 1. We divided the independent variables into 2 categories: those from the decisional needs domains of the Ottawa Decision Support Framework (ODSF)^39^ and other variables (i.e., non-ODSF variables). The ODSF comprises 8 decisional needs domains: 1) decisional type and timing, 2) decisional stage, 3) decisional conflict, 4) knowledge, 5) expectations, 6) values, 7) support and resources to make and implement the decision, and 8) personal and clinical needs.^39^ Based on scientific evidence^40^ and discussions with survey experts and patient partners, which highlighted that the emotional intensity of a consultation has a stronger influence on recall accuracy than the timing of the event, we decided to focus on respondents’ recall of the most difficult decision. Since our data collection was based on a past decision, we added an adjustment variable based on respondents’ self-rated accuracy in recalling a clinical consultation. We measured this variable on a visual analog scale of 0 to 100, where 0 indicated no recall of the consultation and 100 indicated recalling the moment as if the person were still experiencing it. We published the questionnaire with the protocol.^23^

**Table 1:** Independent Variables and Measures.

Construct	Measure	Reference
Independent variables from the Ottawa Decision Support Framework		
Characteristics of the respondents		
Age	Self-developed question driven by the classification used by federal organization (Statistics Canada)	^41^
Education level	Self-developed question driven by the International Standard Classification of Education	^42^
Ethnic and cultural background	Self-developed question driven by the classification used by federal organization (Statistics Canada)	^43^
Gender	Self-developed question driven by the classification used by federal organization (Statistics Canada)	^44^
Geographical area	Postal code including a numeral zero is related to rural area	^45^
Household income	Self-developed question based on a previous survey	^46^
Marital status	Self-developed question driven by the classification used by federal organization (Statistics Canada)	^47^
Pain duration	Self-developed question	
Quality of life	Kemp Quality of Life Scale	^48^
Sex at birth	Self-developed question based on a systematic review	^49^
Spirituality	Self-developed question driven by the classification used by federal organization (Statistics Canada)	^50^
Work status	Self-developed question based on a previous survey	^46^
Decisional needs		
Congruence between assumed and preferred role	Control Preferences Scale	^51^
Decisional conflict	Decisional Conflict Scale	^52^
Decision self-efficacy	Self-developed question	
Difficult decision	Self-developed question driven based on previous study and report	^53–55^
Health literacy	One item of the Brief Health Literacy Screening Tool	^56^
Others involvement	Self-developed question	
Perception of assumed role	Control Preferences Scale	^51^
Prior knowledge	Self-developed question	
Independent variables for the exploratory analysis		
Characteristics of the respondents		
Canadian provinces	Self-developed question driven by the classification used by federal organization (Statistics Canada)	^57^
Comorbidity	Self-developed question based on the chronic pain series from the *Lancet* journals	^58^
First learned language	Self-developed question based on the classification used by federal organization (Statistics Canada)	^59^
Number of people living at home	Self-developed question based on a previous survey	^46^
Pain location	Self-developed question based on the anatomical regions of the body	
Perception of disability and/or emotional distress	Self-developed question based on the IASP definition of high-impact chronic pain	^60^
Perception of stress during the consultation	Self-developed question	
Satisfaction with current health state	Patient Acceptable Symptom State	^61^
Decisional needs		
Congruence between the chosen and preferred option	Self-developed question	
Considered elements	Self-developed question based on the practical issues to inform shared decision-making	^62^
Family burden	Self-developed question based on a previous article	^63^
Treatment burden	Self-developed question based on a previous article	^64^
Adjustment variable		
Accuracy of the consultation recall	Self-developed question	

#### Most difficult decisions (i.e., decisional type of the ODSF)

Based on Canadian reports,^53,54,65^ the study by Poitras et al.,^55^ and the experiences of the patient partners, we proposed 10 prespecified most difficult decisions: 1) Should I take medication or not? 2) Should I get surgery or not? 3) Should I change my treatment? 4) Should I stop my treatment? 5) Should I change my lifestyle habits and behaviors? 6) Should I consult a rehabilitation professional? 7) Should I consult a complementary and alternative medicine professional? 8) Should I consult a mental health professional? 9) Should I change the health care provider to manage my condition? and 10) Should I undergo more diagnostic tests? Respondents could also specify another most difficult decision. We gathered data on the decision making related to the selected most difficult decision.

### Pretesting of the Survey, Administration, and Data Quality

We conducted a content validity evaluation with a clinical sensibility testing^66,67^ involving our patient partners (*n* = 2), experts in survey methodology (*n* = 7), and shared decision-making experts (*n* = 9) and a pilot test with 50 random respondents. After this pretesting, Léger Marketing sent an email invitation to the random samples of eligible participants. Participants had access to the questionnaire for 3 wk. A reminder was sent weekly to solicited participants. All participants completed the questionnaire once. The participation was free and voluntary, but participants received a standardized compensation from Léger Marketing as compensation for the time required to complete the survey. This compensation consists of 1,200 LEO points from Léger Marketing, which could be redeemed for gift cards or a prepaid VISA/Mastercard or donated to charity. To minimize missing data, participants had to respond to all questions to validate and send back the questionnaire.

To ensure data quality, we randomized the order of certain questions within domains to limit question effects.^68^ We randomized the order of the response options when we proposed a list of alternatives to limit response order effects.^68^ Participants were able to stop and save the questionnaire at any time and resume later to limit the loss of accuracy due to fatigue^69^ or due to pain increasing because of the cognitive activity or prolonged seated posture.^70,71^ We removed the questionnaires with a time to complete less than 10 min to limit the loss of accuracy due to questionnaire length.^72^ To identify and remove poor responses, we included quality control questions throughout the survey. These questions asked participants to select a particular response option to make sure they were carefully reading the question and response options. For example, “to make sure we are capturing your responses correctly, please select ‘I don’t know’.”

### Ethical Considerations

We obtained ethics approval from the Research Ethics Board of the Research Centre at the Centre Hospitalier Universitaire de Sherbrooke (project #2022-4645) and respected Canadian regulation on personal information protection. All respondents gave their written informed consent to conduct the study, use any personal information, and publish the study. Data have been anonymized.

### Statistical Analysis

We performed the statistical analyses in R (version 4.3.3, packages reported in Supplementary Material 1). We were supported by biostatisticians for the development of the analyses plan, analysis code, and for the reviewing of the output.

#### Sample size calculation

We targeted a sample size of 1,649 respondents for this survey (see the protocol for details^23^).

#### Data preparation

We estimated coverage and participation biases using data from the 2021 census by Statistics Canada^73^ to determine the need for weighting across provinces. Two independent reviewers performed the initial data analysis (i.e., meta-data, data cleaning, and data screening)^74^ to enhance its robustness and addressed missing data according to the Treatment and Reporting of Missing Data in Observational Studies framework.^75^ We used multiple imputation with multivariate imputation by chained equations (MICE).^76–78^ We report missing data and imputation details in Supplementary Material 1.

#### Characteristics of the respondents

We performed descriptive analysis (mean and standard deviation [*s*], frequency and percentage) for the entire sample, for people with a DRS score = 0 (i.e., absence of decision regret), for people with a DRS score >0 (i.e., presence of decision regret), and for people with a DRS score >25 (i.e., present of important decision regret).

#### Decision regret

We presented the prevalence of decision regret by reporting the percentage of people with a DRS score >0 and also reported the prevalence of important decision regret (DRS score >25). We reported the mean DRS score for each most difficult decision.

#### Multilevel linear regression analysis

We used multilevel linear regression analysis to identify factors associated with decision regret. Based on the ODSF, we developed a descriptive model^79^ that constrained our methodological choices to minimize bias in the regression coefficients.^80^ The ODSF did not allow us to construct a directed acyclic graph; thus, inference should be performed in the global model.^80^ We conducted multilevel analysis to account for potential clustering effects of the Canadian provinces.^81^ We treated level 1 independent variables as fixed effects and the level 2 independent variable (i.e., Canadian provinces) as a random effect.^82^ We visually verified the model assumptions, including outliers, leverage, linearity, normality of residuals, and homoscedasticity.^83^ We provide information on variable recoding and collinearity diagnostics in Supplementary Material 1.

To quantify the total variance explained by the clustering structure, we built an intercept-only model to estimate the intraclass correlation coefficient (ICC).^81^

To identify the factors associated with decision regret, we built a multilevel linear regression model including the variables from the ODSF expanded with the non-ODSF variables (cf. Table 1). We performed regression analysis on each imputed dataset (*n* = 6) and pooled the results. We reported the results with regression coefficients and related 95% confidence intervals. We performed a complete case sensitivity analysis of the model to test the impact of multiple imputation on the results.

## Results

### Survey Administration and Attrition

We randomly invited 31,545 members to Leger Marketing’s panel. The invitation view rate was 18.9% (5,949/31,545). The participation rate (i.e., ratio of unique visitors who agreed to participate/unique first survey page visitors^84^) was 44.8% (2,666/5,949), and the completion rate (i.e., ratio of users who finished the survey/users who agreed to participate^84^) was 61.9% ([1,649/2,666] – 1,649 people living with chronic pain completed the survey in 4 wk from August 31, 2022, to September 28, 2022). We provide a flow diagram of respondent recruitment in Supplementary Material 2. We had no a priori information on the percentage of people living with chronic pain who did not experience difficult decisions (i.e., attrition). After data collection, 276 respondents (16.7%) did not report a most difficult decision, leading to no information on decision regret; 1,373 reported a DRS score.

### Characteristics of the Respondents

We describe the respondents’ characteristics in Supplementary Material 3. Of the 1,373 respondents who experienced a most difficult decision, the mean age was 52 y (*s* = 16.4 y). Half of the respondents were men (49.5%), most lived in urban areas (88.2%), and their pain duration ranged from 3 mo to 59 y. Only 2 respondents (0.2%) had an Aboriginal first language. Five percent perceived assuming a passive role during the decision-making process, and 49.9% of respondents perceived assuming a role that is congruent with their preferred one. Seventy-one percent reported having difficulty understanding what health care providers said about their medical condition, and 2.2% had a low education level (i.e., less than a high school diploma). More than half (56.7%) of the participants involved significant others, including 12.3% who specifically included friends, in their decision-making process. Regarding the family burden, 9.6% of respondents reported that the chosen option had negative financial influence, 9.5% reported negative social influence, while 17.4% found positive influence.

### Decision Regret

**Figure 1:**
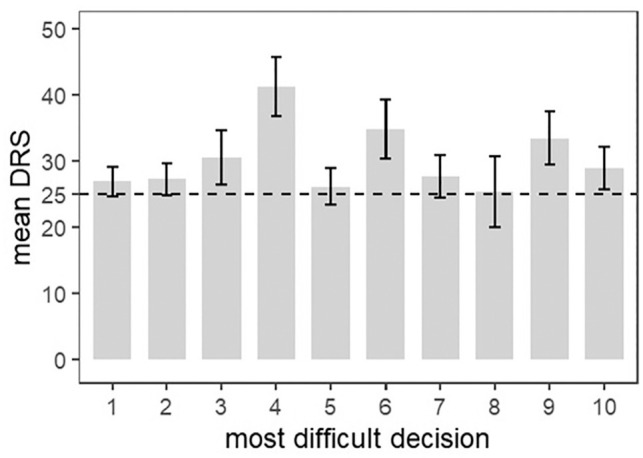
Decision Regret Scale mean score and 95% confidence interval for each most difficult decision. 1: Should I take medication or not?; 2: Should I get surgery or not?; 3: Should I change my treatment?; 4: Should I stop my treatment?; 5: Should I change my lifestyle habits and behaviors?; 6: Should I consult a rehabilitation professional?; 7: Should I consult a complementary and alternative medicine professional?; 8: Should I consult a mental health professional?; 9: Should I change the health care provider to manage my condition?; 10: Should I undergo more diagnostic tests? DRS, Decision Regret Scale.

Respondents had an average decision regret score of 28.8 (*s* = 19.6). Of the 1,373 respondents, 84.3% displayed decision regret (i.e., DRS score >0) and 49.7% had an important level of decision regret (i.e., DRS score >25). We report the details on the DRS score in Table 2 and the distribution of the DRS scores in Supplementary Material 4. Figure 1 presents the mean DRS score for each most difficult decision. All most difficult decisions had a DRS score higher than the cutoff of 25. The most difficult decision with the highest DRS score was “Should I stop my treatment?” (mean DRS score = 41.2 [*s* = 17.3]). We provide more descriptive results in Supplementary Material 4.

**Table 2:** Decision Regret Scale Score and Prevalence.

Decision Regret Scale	
Mean [95% CI]	28.78 [27.76; 29.84]
*s*	19.63
Min	0
Max	100
Median	25
Q1	15
Q3	45
Number of respondents with a DRS score >0	*n* = 1,157
Percentage of respondents with a DRS score >0	84.27%
Number of respondents with a DRS score >25	*n* = 683
Percentage of respondents with a DRS score >25	49.75%

95% CI, 95% confidence interval; DRS, Decision Regret Scale; Q1, first quartile; Q3, third quartile; *s*, standard deviation.

### Multilevel Regression Analysis

From the intercept-only model, we obtained an ICC of 0.012, indicating that a negligeable proportion of the total variance was explained by the clustering structure.

### Factors Associated with Decision Regret

We present the results of the model in Table 3. Of 56 independent variables, we found 15 factors associated with decision regret, of which 10 were associated with an increased risk decision regret and 5 were associated with a reduced risk.

**Table 3:** Regression Coefficients of the Multilevel Linear Model

Variable	Beta	95% CI
Personal characteristics		
Age (per *s* unit)^a^	−1.4	[−2.5; −0.2]
Cultural and ethnical backgrounds		
North American	REF	REF
European	0.2	[−1.8; 2.1]
Asian	3.2	[−0.2; 6.6]
Aboriginal	3.3	[−0.4; 7.0]
Other	4.3	[−0.3; 8.8]
Prefer not to say^a^	7.1	[2.1; 12.0]
Education level		
Above the bachelor’s level	REF	REF
Bachelor’s degree	−0.9	[−3.6; 1.8]
University diploma below bachelor’s level	2.4	[−1.0; 5.7]
College, CEGEP, or other nonuniversity diploma	1.8	[−0.9; 4.5]
High school or equivalency	0.9	[−2.0; 3.9]
Less than high school or equivalency^a^	10.3	[4.3; 16.3]
Geographical area		
Urban	REF	REF
Rural	0.07	[−2.5; 2.6]
Household income (in CAD)		
Less than 50,000	REF	REF
50,000 to less than 60,000	1.6	[−1.1; 4.3]
60,000 to less than 80,000	2.1	[−0.6; 4.8]
80,000 to less than 100,000	2.0	[−0.8; 4.7]
100,000 or more	0.7	[−1.8; 3.2]
Pain duration (per *s* unit)	−0.5	[−1.4; 0.4]
Quality of life	−0.2	[−0.9; 0.6]
Sex		
Male	REF	REF
Female	−1.6	[−3.3; 0.2]
Intersex	0.8	[−14.1; 15.7]
Religious or spiritual affiliation		
No religious or spiritual affiliation	REF	REF
Christian	−0.9	[−3.7; 2.0]
Other religion or spiritual affiliation	−1.4	[−3.2; 0.4]
Perceived disability and/or emotional distress		
Yes	REF	REF
No	−0.4	[−2.1; 1.3]
Health state satisfaction		
Yes	REF	REF
No^a^	4.0	[2.1; 5.9]
Comorbidity		
No comorbidity	−1.8	[−4.4; 0.7]
Diabetes	−0.8	[−3.2; 1.6]
Mental health disorders	−2.5	[−4.7; 0.4]
Alcohol-related disorders	2.8	[−1.7; 7.2]
Substance-related disorders	0.5	[−3.8; 4.7]
Sleep disorders	−0.1	[−2.0; 1.8]
Hypertension	0.8	[−1.3; 2.9]
Disease of the respiratory system	1.7	[−0.7; 4.0]
Other	0.7	[−1.9; 3.4]
Marital status		
Married/living common law	REF	REF
Never married	−0.9	[−3.2; 1.5]
Separated/divorced/widowed	−0.1	[−2.6; 2.4]
Number of people in the household	0.4	[−0.4; 1.1]
First learned language		
English	REF	REF
French	−1.5	[−3.8; 0.8]
An Aboriginal language^a^	24.0	[3.4; 44.7]
Other	0.8	[−2.3; 3.8]
Pain location		
Head or face	−0.2	[−2.4; 2.1]
Abdominal	−1.6	[−4.4; 1.3]
Chest	2.5	[−1.2; 6.2]
Limbs (upper or lower)	−1.0	[−2,7; 0,8]
Back	−1.3	[−3.2; 0.6]
Pelvic	0.5	[−2.1; 3.0]
Decision-making characteristics		
Most difficult decision		
Take medication	REF	REF
Get surgery	0.1	[−2.6; 2.8]
Change treatment	1.1	[−2.3; 4.5]
Stop my treatment^a^	8.0	[3.7; 12.1]
Change lifestyle habits and behaviors	−1.6	[−4.5; 1.3]
Consult a rehabilitation professional	1.9	[−2.3; 6.1]
Consult a CAM professional	−0.1	[−3.2; 3.0]
Consult a mental-health professional	−3.9	[−8.4; 0.7]
Change the health care provider	−1.0	[−4.6; 2.7]
Undergo more diagnostic tests	−1.5	[−4.6; 1.6]
Other	10.8	[−6.2; 27.8]
Prior knowledge on the options		
On all the options	REF	REF
On certain options	−1.4	[−3.4; 0.6]
No prior knowledge^a^	−3.2	[−5.9; −0.5]
Decision self-efficacy	−0.4	[−0.9; 0.1]
Health literacy (problem understanding what doctor says)		
Never	REF	REF
Occasionally	1.3	[−0.8; 3.4]
Sometimes	1.9	[−0.3; 4.1]
Often^a^	6.0	[3.0; 9.0]
Always	4.3	[−1.7; 10.3]
Congruence between chosen and preferred option		
Yes	REF	REF
No^a^	3.4	[0.8; 6.0]
I don’t know, I let my health care professional decide for me	2.0	[−0.2; 4.3]
Perceived stress during the consultation		
No	REF	REF
Yes	−1.1	[−2.9; 0.8]
Decisional conflict (per *s* unit)^a^	8.9	[7.7; 10.0]
Involvement of important other(s)		
Nobody	−1.2	[−3.2; 0.8]
Family	−0.6	[−3.2; 2.0]
Friend^a^	2.8	[0.2; 5.4]
Professional occupation in religion	2.5	[−2.1; 7.2]
Other	−0.6	[−13.7; 12.4]
Perception of assumed decision role		
I made the decision alone	REF	REF
I made the decision alone but considered the opinion of my health care provider	−0.4	[−2.5; 1.7]
My health care providers and I decided together, equally	0.4	[−1.9; 2.6]
My health care providers made the decision but considered my opinion	−0.97	[−4.1; 2.1]
My health care providers made the decision alone^a^	5.3	[1.4; 9.3]
Congruence between assumed and preferred role decision		
No	REF	REF
Yes^a^	−2.2	[−3.9; −0.6]
Considered elements during the decision-making process		
Option’s cost	−0.4	[−2.2; 1.5]
Pressure from others	1.3	[−1.6; 4.2]
Time to implement the option	−0.4	[−2.7; 1.8]
Time before potential outcome	0.2	[−1.8; 2.2]
Delay in accessing the option	0.1	[−2.2; 2.4]
Potential benefits^a^	−1.9	[−3.6; −0.1]
Potential harms	−0.1	[−1.9; 1.7]
Option’s consequences on your social, familial, or affective life	−1.1	[−3.1; 0.9]
Option’s consequences on your diet and consumptions	−1.4	[−3.7; 0.8]
Option’s consequences on a potential pregnancy	2.7	[−1.5; 7.0]
Option’s consequences on your leisure	0.4	[−1.8; 2.4]
Option’s consequences on your work or occupation	0.4	[−1.7; 2.4]
Option’s consequences on your mobility	−0.6	[−2.5; 1.4]
Environmental impacts of the option	−0.4	[−4.5; 3.6]
Other	−1.9	[−10.6; 6.8]
Influences of the chosen option		
Influence of the chosen option on the treatment burden		
No supplementary workload	REF	REF
Less workload	−0.8	[−3.2; 1.6]
Low overload	−0.6	[−2.6; 1.4]
Moderate overload	−0.4	[−2.9; 2.0]
High overload	3.6	[−0.8; 7.9]
Influence of the chosen option on the family		
Influence on daily activities	1.7	[−1.2; 4.6]
Influence on household	−0.60	[−2.8; 1.6]
My family had to spend more time listening to my concerns	−0.20	[−2.6; 2.2]
Negative impacts on the family interaction^a^	4.2	[1.3; 7.0]
Negative economic implications^a^	4.2	[1.3; 7.0]
Positive influence^a^	−5.3	[−7.4; −3.1]
Other	12.2	[−0.8; 25.1]
Adjustment variable		
Accuracy of the recall of the consultation	0.1	[−0.1; 0.1]

a Statistically significant.

95% CI, 95% confidence interval; CAD, Canadian dollar; CAM, complementary and alternative medicine; *s*, standard deviation.

### Factors Associated with Increased Decision Regret

From the multilevel regression analysis, we found 10 variables statistically associated with increasing risk of decision regret (a β shows the expected change in the decision regret per unit increase or between categories, holding other variables constant): 1, having an Aboriginal first language (β = 24.0, 95% CI [3.4; 44.7]) compared with English; 2, having an education level less than a high school diploma (β = 10.3, 95% CI [4.3; 16.3]) compared with above a bachelor’s level; 3, higher decisional conflict (standardized β = 8.9, 95% CI [7.7; 10.0]); 4, decision about stopping the current treatment (β = 7.9, 95% CI [3.7; 12.1]) compared with a decision about taking medication; 5, often having difficulty understanding what health care providers said about their condition (β = 6.0, 95% CI [3.0; 9.0]) compared with never having difficulty; 6, perception of assuming a passive role during the decision-making process (β = 5.3, 95% CI [1.4; 9.3]) compared with an active role; 7, the chosen option having a negative influence on the family (“negative influence on the interaction, routine, leisure, and social network”: β = 4.2, 95% CI [1.3; 7.0], “negative economic implications”: β = 4.2, 95% CI [1.3; 7.0]); 8, being unsatisfied by their current health state (β = 4.0, 95% CI [2.1; 5.9]) compared with being satisfied; 9, no congruence between the chosen and preferred option (β = 3.4, 95% CI [0.8; 6.0]) compared with congruence; and 10, presence of friends during the consultation (β = 2.8, 95% CI [0.2; 5.4]).

### Factors Associated with Reduced Decision Regret

From the multilevel regression analysis, 5 variables were associated with a reduced decision regret (a β shows the expected change in the decision regret per unit increase or between categories, holding other variables constant): 1, the chosen option having a positive influence on the family (β = −5.3, 95% CI [−7.4; −3.1]); 2, having no prior knowledge on the available options (β = −3.2, 95% CI [−5.9; −0.5]) compared with prior knowledge on all options; 3, congruence between the assumed and preferred role during the consultation (β = −2.2, 95% CI [−3.9; −0.6]) compared with noncongruence; 4, considering the benefits of the available options during the decision-making process (β = −1.9, 95% CI [−3.6; −0.1]); and 5, higher age (standardized β = −1.4, 95% CI [−2.5; −0.2]).

### Sensitivity Analysis

We found similar results when comparing the complete case analysis with the imputed analysis, indicating that multiple imputation did not affect our results. Supplementary Material 5 reported the results of the sensitivity analysis.

## Discussion

This pan-Canadian survey highlights the high prevalence and level of decision regret associated with difficult decisions for pain care among people living with chronic pain. We observed that 84% of respondents experienced decision regret and 50% at an important level. We identified 15 factors associated with decision regret, including 4 personal and 9 decision-making characteristics, and 2 consequences of the chosen option. Our findings provide an opportunity to enhance decision making in pain care by addressing factors that contribute to decision regret.

We are among the first to report that more than 8 of 10 people living with chronic pain in Canada experience decision regret when they face a decision deemed as the most difficult one. Our results are higher than those reported by a systematic review, including studies in multiple clinical contexts, such as rheumatology and primary care, in which chronic pain consultations are common.^33^ This review found median prevalences of decision regret of 37% and important decision regret of 9% and a mean DRS score of 16.5.^33^ Our higher prevalence of decision regret is unsurprising, as more than 96% of people living with chronic pain in Canada faced at least 1 difficult decision regarding their pain care.^2^ Our results focused on participants’ most difficult decision rather than their most recent one, which may have skewed the findings upward. Nonetheless, the results consistently reveal a trend: a significant number of people living with chronic pain experience regret regarding their decisions. Moreover, our estimated prevalence of decision regret may be underestimated due to our measurement instrument. Decision regret comprises 4 domains: role regret, process regret, option regret, and outcome regret.^5,7^ The Decision Regret Scale measures only option and outcome regret. In chronic pain care, additional role and process regret can be present. Role regret occurs when individuals regret their role in the decision process without regretting the chosen option.^5^ This may be prevalent in our sample as 50% of the respondents reported noncongruence between the assumed and preferred role during the decision-making process.^2^ This lack of roles congruence could also lead to process regret, which involves regretting the decision-making process, including action or inaction or participation level in the decision-making process.^5^ Our result supports expanding current measurement instruments of decision regret to gain stronger understanding of decision regret in chronic pain care. Decision regret is often interpreted as leading to negative consequences,^5,7,33^ suggesting that the chronic pain situation in Canada is concerning. However, some authors highlight the powerful influence of regret in changing future decision-making behaviors.^5^ The high prevalence of decision regret in chronic pain care could be seen as an opportunity to improve people’s involvement in their health-related decisions.

The decision-making process should be adapted to the individual’s health literacy. Our findings indicate that 71% of respondents had difficulty understanding what health care providers communicated about their medical condition, which is associated with increased decision regret. Although the importance of providing health information in an easily understandable format has been recognized for more than a decade, it remains poorly integrated into clinical practice.^85^ Several strategies have been proposed to address this, including prioritizing essential information, using simple language, minimizing medical jargon, and implementing the teach-back method.^86,87^ These approaches generally place the responsibility on clinicians for patient education. However, time constraints pose a significant barrier to effective information exchange.^88^ A potential solution is preconsultation education using reliable websites or educational materials. However, the quality and readability of these materials often fall short, particularly for individuals with low health literacy.^89,90^ Clinicians are encouraged to assess the readability of these materials before recommending them, using tools such as the Health Literacy Editor software.^85^ Another emerging solution is the use of preconsultation education powered by large language models (e.g., ChatGPT). Studies have demonstrated that these models can provide accurate educational information at a sixth- to seventh-grade reading level^91–94^—a recommended level for health education tools.^95^ For instance, ChatGPT-4 achieved a 96.1% appropriateness rate in responding to 26 guideline-based questions on chronic low-back pain.^94^ However, we found that preconsultation knowledge is associated with higher decision regret. We hypothesize that individuals without prior knowledge may rely solely on structured information from health care providers, reducing cognitive overload and minimizing regret linked to alternative outcomes (i.e., outcome regret).^96^ In this context, preconsultation education could be valuable if it provides reliable, need-specific information without overwhelming the individual. To further streamline the decision-making process, clinicians should prioritize conveying 3 key messages during consultations.^97^ Implementing new technologies in clinical practice could help adapt information to each individual’s information needs and health literacy level.

Treatment discontinuation should be a clear and explicit option within the decision-making process. Our findings indicate that contemplating the decision to stop treatment is associated with greater decision regret. However, we currently lack data to ascertain whether this regret stems from the choice to stop treatment or, conversely, from a decision to continue it. Several factors may explain why individuals consider discontinuing treatment but ultimately choose not to: 1) treatment discontinuation is frequently perceived as a potential loss of therapeutic opportunity^98^; 2) individuals may overestimate the benefits of alternative treatments, leading them to opt for switching therapies rather than complete discontinuation^99^; 3) reluctance to express a desire to stop treatment may arise due to concerns about being perceived as noncompliant or “difficult”^100^; and 4) clinicians’ intervention bias may further discourage consideration of treatment discontinuation.^101^ To our knowledge, no decision aid tool for chronic pain currently presents information on the natural progression of the condition without any treatment. Implementing prognostic models within decision-making interventions could address this gap by predicting future outcomes in the absence of intervention.^102^ For instance, informing an individual that they have a 76% likelihood of quality-of-life improvement over the next 3 mo without treatment could enable a more informed weighing of the pros and cons of initiating medication. Nonetheless, there remains a critical need for externally validated prognostic models, as none are yet deemed clinically valuable in the context of chronic pain.^103^

Matching the assumed and preferred roles seems to be a better way for meeting people’s decisional needs. We found that congruence between assumed and preferred roles is associated with lower decision regret. This finding aligns with recent research, which indicates that role congruence is more influential than the specific role itself in minimizing decision regret.^104^ Not all respondents in this survey wanted the same role in the decision-making process, as 76% of them desired a collaborative role. Imposing a collaborative approach indiscriminately may place greater responsibility on individuals than they desire, potentially increasing decision regret rather than preventing it.^105^ It is essential for clinicians to discuss and identify the role individuals want to assume in the decision-making process. Some personal characteristics, such as lower educational attainment, may result in reluctance to engage actively, often due to limited decision self-efficacy rather than a true preference for noninvolvement.^106^ With adequate information and support, individuals are more likely to participate actively in decision making.^107^ Since assuming a passive role can be linked to greater decision regret, clinicians should first ensure individuals receive sufficient information on the importance of engagement and the type of information addressed in the decision-making process.^108^ Clinicians should also reassess role preferences at each decision point, as preferences can evolve over time.^109^ For example, individuals may experience emotional overload at the initial diagnosis, limiting their capacity to engage in decision making.^110^ As they gain a deeper understanding of their condition, their willingness to engage may increase.^110^ Although only a few clinical shared decision-making models incorporate this step,^111^ assessing a preferred role appears relevant to reducing decision regret.

Considering the impacts of the chosen option on the family could help to reduce decision regret. This study is among the first to show the association between family burden and decision regret. Positive influence on the family, like increased household income due to return to work, is associated with a decreased decision regret, while some negative influences increase it. Among these negative influences, economic and social impacts of the chosen option on the family are associated with increased decision regret. The option’s cost can have negative economic influences and is related to subjective financial toxicity. Subjective financial toxicity refers to people’s perceived distress arising from the cost of the treatment and affecting their well-being and health-related quality of life.^112^ Recently, some authors called to include cost information in SDM interventions.^113,114^ We also argue that clinicians could, pending its availability, incorporate this new element in their consultations regardless of the use of SDM interventions.^114^ Negative influences on social life are also associated with higher decision regret. We found that 80% of respondents reported no to minimal impact of the chosen option on the workload of health care (i.e., treatment burden^115^). This suggests that discussion on the feasibility of the chosen option should extend beyond treatment burden.

The involvement of certain significant others may contribute to increased decision regret. Our findings suggest that the involvement of friends is associated with heightened decision regret, whereas family members or spiritual advisors have no impact. The literature on the role of significant others in decision making is mixed. For example, a recent scoping review notes that relatives can offer both supportive roles (e.g., emotional support, interpretation) and complicating influences (e.g., pressure toward specific treatments).^110^ However, this review does not distinguish between categories such as family, friends, or spiritual advisors. We hypothesize that caregiving roles may partly explain the differing impacts of friends versus family on decision regret. In chronic pain management, informal caregiving responsibilities are typically undertaken by family members, not friends, due to the ongoing support required.^116,117^ Family caregivers frequently provide critical logistical, emotional, and medical support, including transportation to appointments, assistance during treatment side effects, and encouragement for adherence to care plans.^118^ This direct involvement places family members in a unique position to appreciate the complexities of pain management decisions and actively support individuals throughout the decision-making process. In contrast, involving friends in decision making may introduce biases or unintended pressures, as friends often lack the same caregiving responsibilities and comprehensive understanding of the individual’s health needs. This mismatch can create discord between friends’ advice and the practical realities managed by family caregivers, potentially exacerbating family burdens associated with the chosen option, a factor linked to greater decision regret in our survey. Future research could explore whether the inclusion of informal caregivers, rather than generic categories, in decision making correlates with lower decision regret.

### Strengths and Limitations

A strength of our study is the limited risk of coverage and participation biases due to stratified proportional random sampling, improving the representativeness to the Canadian population. A second strength is the use of strategies such as quality control questions to reduce incentive bias. Our sampling underrepresents Canadians from territories and the 6% of the Canadians without home Internet access.^119^ Future studies could replicate our methods in Indigenous people and other hard-to-reach populations, especially considering our result on Aboriginal first learned language. However, with only few respondents with an Aboriginal first language, this result should be interpreted with caution. Another limitation is the retrospective cognitive processes for recall-related questions of a clinical consultation. We controlled for recall bias by adjusting the model with respondent’s perceived recall accuracy. Concerning sample size, not all 1,649 respondents reported a Decision Regret Scale score. We compared the characteristics of the 1,649 and the 1,373 respondents to verify that attrition did not affect representativeness. From the initial model, we found a post hoc calculation of 24.5 events per variable, exceeding the stringent rule of 15.^80,120^ A limitation of this study is that we collected only broad categories of options without requesting recall of every specific option within each category, due to feasibility concerns and potential issues with recall validity. Achieving this level of detail would require linkage with provincial databases, which is not feasible across all 10 provinces in Canada. This approach does not enable the identification of specific options that may drive higher levels of regret (e.g., surgery). Lastly, as this is the first pan-Canadian survey on this topic, various questions were newly formulated. We improved the content validity through a face validity process with patients and experts.

## Conclusion

This pan-Canadian survey highlights the high prevalence of decision regret associated with difficult decisions for pain care. Eighty-four percent experienced decision regret, and 50% of our sample had important decision regret. We identified 15 factors associated with decision regret. Among those, 4 factors were personal characteristics, 9 were decision-making characteristics, and 2 were consequences of the chosen option. Decision making in pain care could be enhanced by addressing factors that contribute to decision regret.

## Supplemental Material

sj-docx-1-mdm-10.1177_0272989X251326069 – Supplemental material for People Living with Chronic Pain Experience a High Prevalence of Decision Regret in Canada: A Pan-Canadian Online Survey Supplemental material, sj-docx-1-mdm-10.1177_0272989X251326069 for People Living with Chronic Pain Experience a High Prevalence of Decision Regret in Canada: A Pan-Canadian Online Survey by Florian Naye, Yannick Tousignant-Laflamme, Maxime Sasseville, Chloé Cachinho, Thomas Gérard, Karine Toupin-April, Olivia Dubois, Jean-Sébastien Paquette, Annie LeBlanc, Isabelle Gaboury, Marie-Ève Poitras, Linda C. Li, Alison M. Hoens, Marie-Dominique Poirier, France Légaré and Simon Décary in Medical Decision Making

sj-docx-2-mdm-10.1177_0272989X251326069 – Supplemental material for People Living with Chronic Pain Experience a High Prevalence of Decision Regret in Canada: A Pan-Canadian Online Survey Supplemental material, sj-docx-2-mdm-10.1177_0272989X251326069 for People Living with Chronic Pain Experience a High Prevalence of Decision Regret in Canada: A Pan-Canadian Online Survey by Florian Naye, Yannick Tousignant-Laflamme, Maxime Sasseville, Chloé Cachinho, Thomas Gérard, Karine Toupin-April, Olivia Dubois, Jean-Sébastien Paquette, Annie LeBlanc, Isabelle Gaboury, Marie-Ève Poitras, Linda C. Li, Alison M. Hoens, Marie-Dominique Poirier, France Légaré and Simon Décary in Medical Decision Making

sj-docx-3-mdm-10.1177_0272989X251326069 – Supplemental material for People Living with Chronic Pain Experience a High Prevalence of Decision Regret in Canada: A Pan-Canadian Online Survey Supplemental material, sj-docx-3-mdm-10.1177_0272989X251326069 for People Living with Chronic Pain Experience a High Prevalence of Decision Regret in Canada: A Pan-Canadian Online Survey by Florian Naye, Yannick Tousignant-Laflamme, Maxime Sasseville, Chloé Cachinho, Thomas Gérard, Karine Toupin-April, Olivia Dubois, Jean-Sébastien Paquette, Annie LeBlanc, Isabelle Gaboury, Marie-Ève Poitras, Linda C. Li, Alison M. Hoens, Marie-Dominique Poirier, France Légaré and Simon Décary in Medical Decision Making

sj-docx-4-mdm-10.1177_0272989X251326069 – Supplemental material for People Living with Chronic Pain Experience a High Prevalence of Decision Regret in Canada: A Pan-Canadian Online Survey Supplemental material, sj-docx-4-mdm-10.1177_0272989X251326069 for People Living with Chronic Pain Experience a High Prevalence of Decision Regret in Canada: A Pan-Canadian Online Survey by Florian Naye, Yannick Tousignant-Laflamme, Maxime Sasseville, Chloé Cachinho, Thomas Gérard, Karine Toupin-April, Olivia Dubois, Jean-Sébastien Paquette, Annie LeBlanc, Isabelle Gaboury, Marie-Ève Poitras, Linda C. Li, Alison M. Hoens, Marie-Dominique Poirier, France Légaré and Simon Décary in Medical Decision Making

sj-docx-5-mdm-10.1177_0272989X251326069 – Supplemental material for People Living with Chronic Pain Experience a High Prevalence of Decision Regret in Canada: A Pan-Canadian Online Survey Supplemental material, sj-docx-5-mdm-10.1177_0272989X251326069 for People Living with Chronic Pain Experience a High Prevalence of Decision Regret in Canada: A Pan-Canadian Online Survey by Florian Naye, Yannick Tousignant-Laflamme, Maxime Sasseville, Chloé Cachinho, Thomas Gérard, Karine Toupin-April, Olivia Dubois, Jean-Sébastien Paquette, Annie LeBlanc, Isabelle Gaboury, Marie-Ève Poitras, Linda C. Li, Alison M. Hoens, Marie-Dominique Poirier, France Légaré and Simon Décary in Medical Decision Making
