# Supplementary material for: People Living with Chronic Pain Experience a High Prevalence of Decision Regret in Canada: A Pan-Canadian Online Survey
Source: Med Decis Making. 2025 Mar 22;45(4):462–79. doi: 10.1177/0272989X251326069 (PMC11992647; doi:10.1177/0272989X251326069)
Supplement: sj-docx-1-mdm-10.1177_0272989X251326069 – Supplemental material for People Living with Chronic Pain Experience a High Prevalence of Decision Regret in Canada: A Pan-Canadian Online Survey [file sj-docx-1-mdm-10.1177_0272989X251326069.docx]

**Supplementary Material 1**: Detailed information on the methods.

1. **R packages**

| **Purpose** | **Package** |
| --- | --- |
| Data preparation | dplyr  readxl |
| Data visualizations | ggplot2  ggthemes |
| Multiple Imputation by Chained Equations | mice |
| Multilevel regression | lme4  broom.mixed  performance |
| Missing data | performance  dplyr |
| Sample size | pmsampsize |

1. **Missing data and multiple imputation**

Missing data could be observed due to data cleaning, “prefer not to say” response option, and “I don’t remember” response option. We assumed that the missing data were Missing At Random (MAR).

**Distribution of the missing data:**

We observed missing data on eleven independent variables ranged between 9.83% (pain duration) and 0.07% (sex) of missing data.

We observed missing data on 357 (26%) observations ranged between 6.93% and 0.99% of missing data.

**Imputation technique:**

We used predictive mean matching method as imputation technique because it is an effective imputation approach for continuous, ordered categorical and dichotomous multilevel data (1). Independent variables with collinearity (see below) were deleted of the imputation model (2).

**Imputation model:**

We used all the variables (dependent and independent) of the initial model except gender, work status, number of comorbidities, and number of pain location because of their collinearity (VIF>2.5) (3).

We checked the consistency of the imputed data with data visualization (function densityplot of the MICE package).

**Method to calculate the number of imputation cycles:**

We calculated fraction of missing information (i.e., an important parameter for diagnosing the effects of data missingness (4) to determine the number of multiple imputation cycles. The highest fraction of missing information was 0.085 (pain duration variable) leading to six cycles of multiple imputation (5).

**References:**

1.Vink G, Lazendic G, van Buuren S. Partitioned predictive mean matching as a multilevel imputation technique. Psychological Test and Assessment Modeling. 2015;57(577-594).

2.Nguyen CD, Carlin JB, Lee KJ. Practical strategies for handling breakdown of multiple imputation procedures. Emerg Themes Epidemiol. 2021;18(1):5.

3.Johnston R, Jones K, Manley D. Confounding and collinearity in regression analysis: a cautionary tale and an alternative procedure, illustrated by studies of British voting behaviour. Qual Quant. 2018;52(4):1957-76.

4.Madley-Dowd P, Hughes R, Tilling K, Heron J. The proportion of missing data should not be used to guide decisions on multiple imputation. J Clin Epidemiol. 2019;110:63-73.

5.Bodner TE. What Improves with Increased Missing Data Imputations? Structural Equation Modeling: A Multidisciplinary Journal. 2008;15(4):651-75.
