# Supplementary figures and images for: People Living with Chronic Pain Experience a High Prevalence of Decision Regret in Canada: A Pan-Canadian Online Survey
Source: Med Decis Making. 2025 Mar 22;45(4):462–79. doi: 10.1177/0272989X251326069 (PMC11992647; doi:10.1177/0272989X251326069)

**Supplementary Material 2:** Flow chart of respondent recruitment.

**
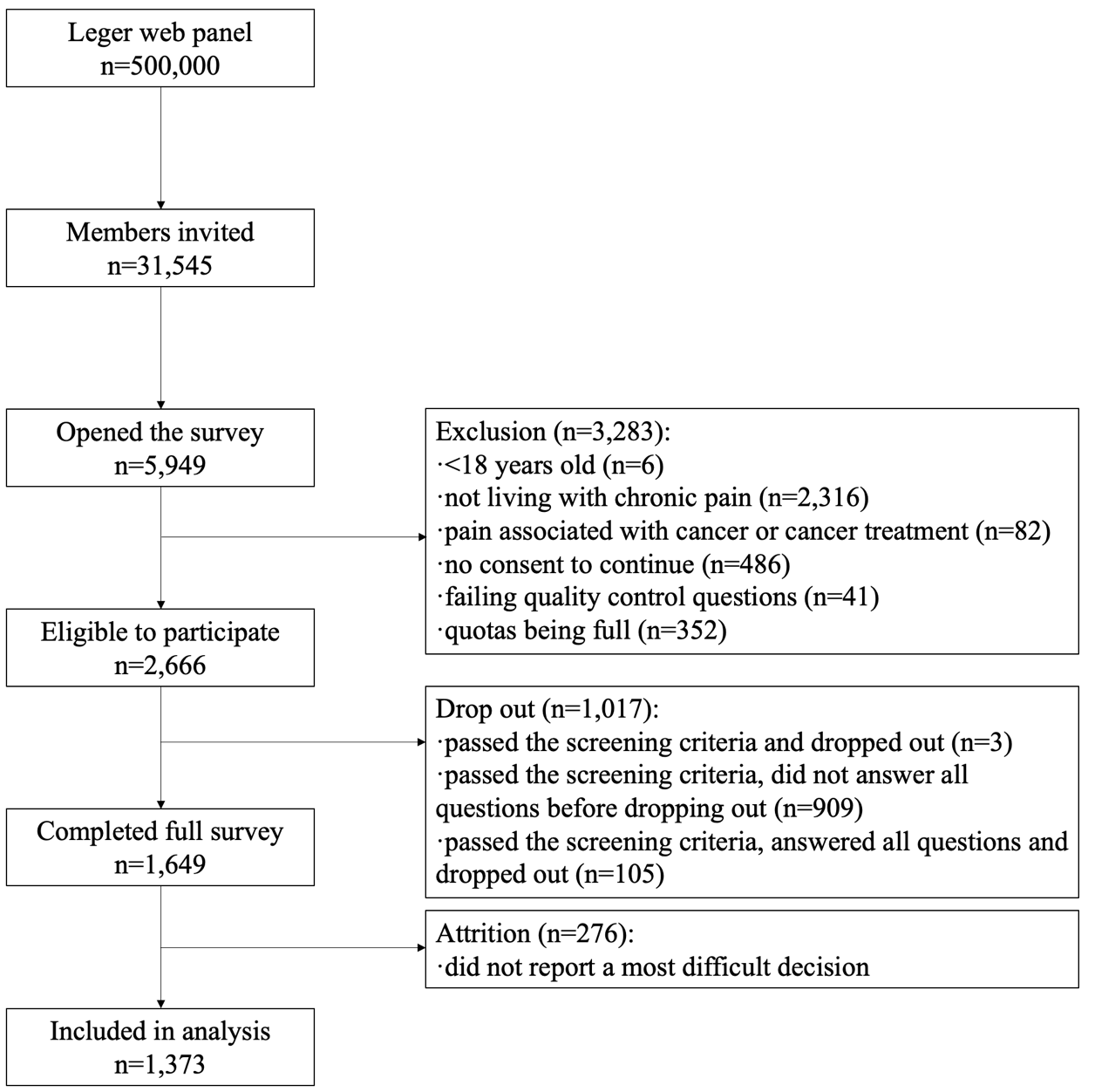
**

Supplement: sj-docx-2-mdm-10.1177_0272989X251326069 – Supplemental material for People Living with Chronic Pain Experience a High Prevalence of Decision Regret in Canada: A Pan-Canadian Online Survey [file sj-docx-2-mdm-10.1177_0272989X251326069.docx]
