# Supplementary material for: People Living with Chronic Pain Experience a High Prevalence of Decision Regret in Canada: A Pan-Canadian Online Survey
Source: Med Decis Making. 2025 Mar 22;45(4):462–79. doi: 10.1177/0272989X251326069 (PMC11992647; doi:10.1177/0272989X251326069)
Supplement: sj-docx-3-mdm-10.1177_0272989X251326069 – Supplemental material for People Living with Chronic Pain Experience a High Prevalence of Decision Regret in Canada: A Pan-Canadian Online Survey [file sj-docx-3-mdm-10.1177_0272989X251326069.docx]

**Supplementary Material 3:** Characteristics of the respondents.

| **Characteristics** | **Descriptive analyses** | **Complete sample (n=1,373)** | | | | **DRS=0 (n=216)** | | | | **DRS>0 (n=1,157)** | | | | **DRS>25 (n=683)** | |  |
| --- | --- | --- | --- | --- | --- | --- | --- | --- | --- | --- | --- | --- | --- | --- | --- | --- |
| **Socio-demographic characteristics** | | | | | | | | | | | | | | | |  |
| Age (years) | Mean | 51.34 | | | | 55.33 | | | | 50.59 | | | | 48.49 | |  |
|  | [95% CI] | [50.47 ; 52.20] | | | | [53.32 ; 57.35] | | | | [46.64 ; 51.54] | | | | [47.24 ; 49.74] | |  |
|  | SD | 16.36 | | | | 15.06 | | | | 16.49 | | | | 16.6 | |  |
|  | Min | 18 | | | | 21 | | | | 18 | | | | 18 | |  |
|  | Max | 90 | | | | 82 | | | | 90 | | | | 90 | |  |
| Sex | Male: n (%) | 686 | | (49.96%) | | 100 | | (46.30%) | | 586 | | (50.65%) | | 351 | (51.39%) |  |
|  | Female: n (%) | 682 | | (49.67%) | | 116 | | (53.70%) | | 566 | | (48.91%) | | 328 | (48.02%) |  |
|  | Intersex: n (%) | 4 | | (0.29%) | | 0 | | (0.00%) | | 4 | | (0.35%) | | 4 | (0.59%) |  |
|  | Prefer not to say: n (%) | 1 | | (0.07%) | | 0 | | (0.00%) | | 1 | | (0.09%) | | 0 | (0.00%) |  |
| Gender | Man: n (%) | 680 | | (49.53%) | | 97 | | (44.91%) | | 583 | | (50.39%) | | 347 | (50.81%) |  |
|  | Woman: n (%) | 671 | | (48.87%) | | 115 | | (53.24%) | | 556 | | (48.05%) | | 321 | (47.00%) |  |
|  | Non-binary: n (%) | 8 | | (0.58%) | | 0 | | (0.00%) | | 8 | | (0.69%) | | 6 | (0.88%) |  |
|  | Transgender woman: n (%) | 1 | | (0.07%) | | 0 | | (0.00%) | | 1 | | (0.09%) | | 1 | (0.15%) |  |
|  | Transgender man: n (%) | 2 | | (0.15%) | | 0 | | (0.00%) | | 2 | | (0.17%) | | 2 | (0.29%) |  |
|  | Two-spirit: n (%) | 3 | | (0.22%) | | 2 | | (0.93%) | | 1 | | (0.09%) | | 1 | (0.15%) |  |
|  | Gender fluid: n (%) | 0 | | (0.00%) | | 0 | | (0.00%) | | 3 | | (0.26%) | | 2 | (0.29%) |  |
|  | Other: n (%) | 3 | | (0.22%) | | 0 | | (0.00%) | | 0 | | (0.00%) | | 0 | (0.00%) |  |
|  | Prefer not to say: n (%) | 0 | | (0.00%) | | 0 | | (0.00%) | | 3 | | (0.26%) | | 0 | (0.00%) |  |
|  | Missing data: n (%) | 5 | | (0.36%) | | 2 | | (0.93%) | | 3 | | (0.26%) | | 3 | (0.44%) |  |
| Provinces | British Columbia: n (%) | 220 | | (16.02%) | | 31 | | (14.35%) | | 189 | | (16.33%) | | 129 | (18.89%) |  |
|  | Alberta: n (%) | 188 | | (13.69%) | | 39 | | (18.06%) | | 149 | | (12.88%) | | 86 | (12.59%) |  |
|  | Prairies: n (%) | 103 | | (7.50%) | | 18 | | (8.33%) | | 85 | | (7.35%) | | 51 | (7.47%) |  |
|  | Ontario: n (%) | 487 | | (35.47%) | | 64 | | (29.63%) | | 423 | | (36.56%) | | 256 | (37.48%) |  |
|  | Quebec: n (%) | 273 | | (19.88%) | | 49 | | (22.68%) | | 224 | | (19.36%) | | 117 | (17.13%) |  |
|  | Atlantic Canada: n (%) | 102 | | (7.43%)) | | 15 | | (6.94%) | | 87 | | (7.52%) | | 44 | (6.44%) |  |
| Geographical area | Rural: n (%) | 162 | | (11.80%) | | 31 | | (14.35%) | | 131 | | (11.32%) | | 70 | (10.25%) |  |
|  | Urban: n (%) | 1,211 | | (88.20%) | | 185 | | (85.65%) | | 1,026 | | (88.68%) | | 613 | (89.75%) |  |
| First learned language | French: n (%) | 315 | | (22.94%) | | 55 | | (25.46%) | | 260 | | (22.47%) | | 135 | (19.77%) |  |
|  | English: n (%) | 931 | | (67.81%) | | 140 | | (64.81%) | | 791 | | (68.37%) | | 473 | (69.25%) |  |
|  | An Aboriginal language: n (%) | 2 | | (0.15%) | | 0 | | (0.00%) | | 2 | | (0.17%) | | 2 | (0.29%) |  |
|  | Spanish: n (%) | 9 | | (0.66%) | | 1 | | (0.46%) | | 8 | | (0.69%) | | 6 | (0.88%) |  |
|  | Mandarin: n (%) | 15 | | (1.09%) | | 3 | | (1.39%) | | 12 | | (1.04%) | | 9 | (1.32%) |  |
|  | Arabic: n (%) | 9 | | (0.66%) | | 1 | | (0.46%) | | 8 | | (0.69%) | | 7 | (1.02%) |  |
|  | Other: n (%) | 92 | | (6.70%) | | 16 | | (7.41%) | | 76 | | (6.57%) | | 51 | (7.47%) |  |
| Education level | Less than a high school diploma: n (%) | 30 | | (2.18%) | | 2 | | (0.93%) | | 28 | | (2.42%) | | 21 | (3.07%) |  |
|  | High School diploma: n (%) | 264 | | (19.23%) | | 44 | | (20.37%) | | 220 | | (19.01%) | | 129 | (18.89%) |  |
|  | College, CEGEP or other non-university certificate or diploma: n (%) | 405 | | (29.50%) | | 72 | | (33.33%) | | 333 | | (28.78%) | | 197 | (28.84%) |  |
|  | University certificate: n (%) | 135 | | (9.83%) | | 713 | | (6.02%) | | 122 | | (10.54%) | | 77 | (11.27%) |  |
|  | Bachelor’s degree: n (%) | 351 | | (25.56%) | | 58 | | (26.85%) | | 293 | | (25.32%) | | 167 | (24.45%) |  |
|  | Above the bachelor’s level: n (%) | 181 | | (13.18%) | | 25 | | (11.57%) | | 156 | | (13.48%) | | 89 | (13.03%) |  |
|  | Prefer not to say: n (%) | 7 | | (0.51%) | | 2 | | (0.93%) | | 5 | | (0.43%) | | 3 | (0.44%) |  |
| Cultural and ethnical backgrounds | Aboriginal: n (%)  Aboriginal – African: n (%)  Aboriginal – Caribbean: n (%)  Aboriginal – European: n (%)  Aboriginal – North American: n (%)  Aboriginal – North American – European: n (%)  Aboriginal – North American – European – Asian: n (%)  African: n (%) | 49  1  1  8  10  4  1  17 | | (3.57%)  (0.07%)  (0.07%)  (0.58%)  (0.73%)  (0.29%)  (0.07%)  (1.24%) | | 6  0  0  2  2  1  0  2 | | (2.78%)  (0.00%)  (0.00%)  (0.93%)  (0.93%)  (0.46%)  (0.00%)  (0.93%) | | 43  1  1  6  8  3  1  15 | | (3.72%)  (0.08%)  (0.09%)  (0.52%)  (0.69%)  (0.26%)  (0.09%)  (1.30%) | | 30  0  1  3  6  1  0  13 | (4.39%)  (0.00%)  (0.15%)  (0.44%)  (0.88%)  (0.15%)  (0.00%)  (1.90%) |  |
|  | African – Asian: n (%)  Asian: n (%)  Asian – Oceanian: n (%)  Caribbean: n (%)  Caribbean – Latin American: n (%)  European: n(%)  European – African: n (%)  European – Asian: n (%)  European – Caribbean: n (%)  European – Caribbean – Latin American: n (%)  European – Latin American: n (%)  European – Oceanian: n (%)  Latin American: n (%)  North American: n (%)  North American – Asian: n (%)  North American – Caribbean: n (%)  North American – European: n (%)  North American – European – Asian: n (%)  Oceanian: n (%)  Other: n (%)  Prefer not to say: n (%) | 1  132  1  13  2  454  1  6  1  1  2  1  10  534  2  2  72  1  1  4  38 | | (0.07%)  (9.61%)  (0.07%)  (0.95%)  (0.14%)  (33.1%)  (0.07%)  (0.44%)  (0.07%)  (0.07%)  (0.14%)  (0.07%)  (0.73%)  (38.9%)  (0.14%)  (0.14%)  (5.24%)  (0.07%)  (0.07%)  (0.29%)  (2.77%) | | 0  12  1  4  0  78  0  1  0  0  0  0  1  87  0  0  15  0  0  0  4 | | (0.00%)  (5.56%)  (0.46%)  (1.85%)  (0.00%)  (36.11%)  (0.00%)  (0.46%)  (0.00%)  (0.00%)  (0.00%)  (0.00%)  (0.46%)  (40.28%)  (0.00%)  (0.00%)  (6.94%)  (0.00%)  (0.00%)  (0.00%)  (1.85%) | | 1  120  0  9  2  376  1  5  1  1  2  1  9  447  2  2  57  1  1  4  34 | | (0.09%)  (10.37%)  (0.00%)  (0.78%)  (0.17%)  (32.50%)  (0.09%)  (0.43%)  (0.09%)  (0.09%)  (0.17%)  (0.08%)  (0.78%)  (38.63%)  (0.17%)  (0.17%)  (4.93%)  (0.08%)  (0.08%)  (0.35%)  (2.94%) | | 1  95  0  9  1  207  0  4  1  0  2  0  7  243  2  1  27  1  0  2  25 | (0.15%)  (13.91%)  (0.00%)  (1.32%)  (0.15%)  (30.31%)  (0.00%)  (0.59%)  (0.15%)  (0.00%)  (0.29%)  (0.00%)  (1.02%)  (35.58%)  (0.29%)  (0.15%)  (3.95%)  (0.14%)  (0.00%)  (0.29%)  (3.66%) |  |
|  | Missing data: n (%) | 3 | | (0.22%) | | 0 | | (0.00%) | | 3 | | (0.26%) | | 1 | (0.14%) |  |
|  | Mean | 1.06 | | | | 1.08 | | | | 1.09 | | | | 1.04 | |  |
|  | [95% CI] | [1.04 ; 1.08] | | | | [1.04 ; 1.14] | | | | [1.07 ; 1.11] | | | | [1.01 ; 1.06] | |  |
|  | SD | 0.36 | | | | 0.32 | | | | 0.31 | | | | 0.28 | |  |
|  | Min | 0 | | | | 1 | | | | 1 | | | | 0 | |  |
|  | Max | 4 | | | | 3 | | | | 4 | | | | 3 | |  |
| Spirituality | Buddhist: n (%) | 17 | | (1.24%) | | 0 | | (0.00%) | | 17 | | (1.47%) | | 12 | (1.76%) |  |
|  | Christian: n (%) | 578 | | (42.10%) | | 107 | | (49.54%) | | 471 | | (40.71%) | | 258 | (37.77%) |  |
|  | Hindu: n (%) | 12 | | (0.87%) | | 0 | | (0.00%) | | 12 | | (1.04%) | | 8 | (1.17%) |  |
|  | Jewish: n (%) | 19 | | (1.38%) | | 2 | | (0.93%) | | 17 | | (1.47%) | | 8 | (1.17%) |  |
|  | Muslim: n (%) | 31 | | (2.25%) | | 4 | | (1.85%) | | 27 | | (2.33%) | | 23 | (3.37%) |  |
|  | Sikh: n (%) | 13 | | (0.95%) | | 0 | | (0.00%) | | 13 | | (1.23%) | | 10 | (1.46%) |  |
|  | Traditional (North American Indigenous) spirituality: n (%) | 21 | | (1.53%) | | 4 | | (1.85%) | | 17 | | (1.47%) | | 10 | (1.46%) |  |
|  | Other: n (%) | 26 | | (1.89%) | | 6 | | (2.78%) | | 20 | | (1.73%) | | 12 | (1.76%) |  |
|  | No religious or spiritual affiliations: n (%) | 601 | | (43.77%) | | 88 | | (40.74%) | | 513 | | (44.33%) | | 305 | (44.66%) |  |
|  | Prefer not to say: n (%) | 54 | | (3.93%) | | 5 | | (2.31%) | | 49 | | (4.24%) | | 37 | (5.42%) |  |
|  | Missing data: n (%) | 1 | | (0.07%) | | 0 | | (0.00%) | | 1 | | (0.09%) | | 0 | (0.00%) |  |
| Marital status | Never legally married: n (%) | 329 | | (23.96%) | | 49 | | (22.68%) | | 280 | | | (24.20%) | 177 | (25.91%) |  |
|  | Legally married: n (%) | 647 | | (47.12%) | | 105 | | (48.61%) | | 542 | | (46.85%) | | 310 | (45.39%) |  |
|  | Separated, but still legally married: n (%) | 40 | | (2.91%) | | 2 | | (0.93%) | | 38 | | (3.28%) | | 24 | (3.51%) |  |
|  | Divorced: n (%) | 116 | | (8.44%) | | 21 | | (9.72%) | | 95 | | (8.21%) | | 55 | (8.05%) |  |
|  | Widowed: n (%) | 50 | | (3.64%) | | 9 | | (4.17%) | | 41 | | (3.54%) | | 22 | (3.22%) |  |
|  | Living common law: n (%) | 170 | | (12.38%) | | 28 | | (12.96%) | | 142 | | (12.27%) | | 79 | (11.57%) |  |
|  | Prefer not to say: n (%) | 21 | | (1.53%) | | 2 | | (0.93%) | | 19 | | (1.64%) | | 16 | (2.34%) |  |
| Number of people in the household | Mean | 2.49 | | | | 2.44 | | | | 2.51 | | | | 2.58 | |  |
|  | [95% CI] | [2.47 ; 2.56] | | | | [2.27 ; 2.61] | | | | [2.43 ; 2.58] | | | | [2.48 ; 2.68] | |  |
|  | SD | 1.28 | | | | 1.24 | | | | 1.29 | | | | 1.35 | |  |
|  | Min | 1 | | | | 1 | | | | 1 | | | | 1 | |  |
|  | Max | 10 | | | | 7 | | | | 10 | | | | 10 | |  |
| Household income (CAD) | Less than $50,000: n (%) | 388 | | (28.26%) | | 71 | | (32.87%) | | 317 | | (27.40%) | | 191 | (27.96%) |  |
|  | $50,000 to less than $60,000: n (%) | 172 | | (12.53%) | | 24 | | (11.11%) | | 148 | | (12.79%) | | 93 | (13.62%) |  |
|  | $60,000 to less than $80,000: n (% | 185 | | (13.47%) | | 22 | | (10.18%) | | 163 | | (14.09%) | | 103 | (15.08%) |  |
|  | $80,000 to less than $100,000: n (%) | 192 | | (13.98%) | | 33 | | (15.28%) | | 159 | | (13.74%) | | 92 | (13.47%) |  |
|  | $100,000 or more: n (%) | 350 | | (25.49%) | | 57 | | (26.39%) | | 293 | | (25.32%) | | 148 | (21.67%) |  |
|  | Prefer not to say: n (%) | 86 | | (6.26%) | | 9 | | (4.17%) | | 77 | | (6.66%) | | 56 | (8.20%) |  |
| Work status | Currently working full-time: n (%) | 551 | | (40.13%) | | 73 | | (33.80%) | | 478 | | (41.32%) | | 291 | (42.61%) |  |
|  | Currently working part-time: n (%) | 123 | | (8.9%) | | 23 | | (10.65%) | | 100 | | (8.64%) | | 53 | (7.75%) |  |
|  | Homemaker, no outside employment: n (%) | 54 | | (3.93%) | | 14 | | (6.48%) | | 40 | | (3.46%) | | 26 | (3.81%) |  |
|  | Student: n (%) | 51 | | (3.71%) | | 3 | | (1.39%) | | 48 | | (4.15%) | | 32 | (4.68%) |  |
|  | Unemployed: n (%) | 52 | | (3.78%) | | 5 | | (2.31%) | | 47 | | (4.06%) | | 33 | (4.83%) |  |
|  | Retired: n (%) | 415 | | (30.23%) | | 79 | | (36.57%) | | 336 | | (29.04%) | | 178 | (26.06%) |  |
|  | Long term or permanent disability: n (%) | 85 | | (6.19%) | | 13 | | (6.02%) | | 72 | | (6.22%) | | 44 | (6.44%) |  |
|  | Total sick leave with financial assistance: n (%) | 15 | | (1.09%) | | 3 | | (1.39%) | | 12 | | (1.04%) | | 9 | (1.32%) |  |
|  | Total sick leave with no financial assistance: n (%) | 11 | | (0.80%) | | 1 | | (0.46%) | | 10 | | (0.86%) | | 6 | (0.88%) |  |
|  | Partial sick leave: n (%) | 3 | | (0.22%) | | 1 | | (0.46%) | | 2 | | (0.17%) | | 1 | (0.15%) |  |
|  | Prefer not to say: n (%) | 13 | | (0.95%) | | 1 | | (0.46%) | | 12 | | (1.04%) | | 10 | (1.46%) |  |
| **Pain characteristics** | | | | | | | | | | | | | | | |  |
| Pain location | Head: n (%) | 213 | | (15.51%) | | 22 | | (10.19%) | | 191 | | (16.51%) | | 121 | (17.72%) |  |
|  | Face: n (%) | 46 | | (3.35%) | | 3 | | (1.39%) | | 43 | | (3.72%) | | 32 | (4.68%) |  |
|  | Neck: n (%) | 478 | | (34.81%) | | 73 | | (33.80%) | | 405 | | (35.00%) | | 241 | (35.28%) |  |
|  | Middle back: n (%) | 409 | | (29.79%) | | 69 | | (31.94%) | | 340 | | (29.39%) | | 211 | (20.89%) |  |
|  | Low back: n (%) | 794 | | (57.83%) | | 129 | | (59.72%) | | 665 | | (57.48%) | | 413 | (60.47%) |  |
|  | Upper limb: n (%) | 337 | | (24.54%) | | 62 | | (28.70%) | | 275 | | (23.77%) | | 161 | (23.57%) |  |
|  | Lower limb: n (%) | 544 | | (39.62%) | | 101 | | (46.76%) | | 443 | | (38.29%) | | 252 | (36.90%) |  |
|  | Abdominal: n (%) | 135 | | (9.83%) | | 19 | | (8.80%) | | 116 | | (10.03%) | | 78 | (11.43%) |  |
|  | Pelvic: n (%) | 163 | | (11.88%) | | 19 | | (8.80%) | | 144 | | (12.45%) | | 94 | (13.76%) |  |
|  | Chest: n (%) | 73 | | (5.32%) | | 8 | | (3.70%) | | 65 | | (5.62%) | | 47 | (6.88%) |  |
|  | Other: n (%) | 9 | | (0.66%) | | 1 | | (0.46%) | | 8 | | (0.69%) | | 4 | (0.59%) |  |
|  | Missing data: n (%) | 1 | | (0.07%) | | 0 | | (0.00%) | | 1 | | (0.08%) | | 1 | (0.15%) |  |
|  | Mean | 2.33 | | | | 2.34 | | | | 2.33 | | | | 2.42 | |  |
|  | [95% CI] | [2.25 ; 2.41] | | | | [2.16 ; 2.53] | | | | [2.41 ; 2.42] | | | | [2.29 ; 2.55] | |  |
|  | SD | 1.53 | | | | 1.39 | | | | 1.56 | | | | 1.68 | |  |
|  | Min | 1 | | | | 1 | | | | 1 | | | | 0 | |  |
|  | Max | 10 | | | | 7 | | | | 10 | | | | 10 | |  |
| Pain duration (months) | Mean | 102.19 | | | | 126.91 | | | | 97.48 | | | | 96.6 | |  |
|  | [95% CI] | [95.83 ; 108.54] | | | | [107.79 ; 146.04] | | | | [90.87 ; 106.09] | | | | [87.74 ; 105.36] | |  |
|  | SD | 113.95 | | | | 136.48 | | | | 108.58 | | | | 110 | |  |
|  | Min | 3 | | | | 3 | | | | 3 | | | | 3 | |  |
|  | Max | 708 | | | | 708 | | | | 636 | | | | 600 | |  |
|  | Missing data: n (%) | 135 | | | (9.83%) | 18 | | | 78 | (11.42%) | | | (10.15%) | 78 | (11.42%) |  |
| Comorbidity | Diabetes: n (%) | 204 | | (14.85%) | | 31 | | (14.35%) | | 173 | | 14.95%) | | 100 | (14.64%) |  |
|  | Mental health disorders: n (%) | 438 | | (31.90%) | | 62 | | (28.70%) | | 376 | | 32.50%) | | 224 | (32.80%) |  |
|  | Alcohol-related disorders: n (%) | 49 | | (3.57%) | | 5 | | (2.31%) | | 44 | | (3.80%) | | 36 | (5.27%) |  |
|  | Substance-related disorders: n (%) | 54 | | (3.93%) | | 6 | | (2.78%) | | 48 | | (4.15%) | | 38 | (5.56%) |  |
|  | Sleep disorders: n (%) | 417 | | (30.37%) | | 71 | | (32.87%) | | 346 | | (29.90%) | | 220 | (32.31%) |  |
|  | Hypertension: n (%) | 373 | | (27.17%) | | 62 | | (28.70%) | | 311 | | (26.88%) | | 182 | (26.65%) |  |
|  | Respiratory disorders: n (%) | 193 | | (14.06%) | | 26 | | (12.03%) | | 167 | | (14.43%) | | 102 | (14.93%) |  |
|  | Other: n (%) | 178 | | (12.96%) | | 32 | | (14.81%) | | 146 | | (12.62%) | | 79 | (11.57%) |  |
|  | No comorbidity: n (%) | 360 | | (26.22%) | | 65 | | (30.09%) | | 295 | | (25.50%) | | 161 | (23.57%) |  |
|  | Mean | 2.38 | | | | 2.36 | | | | 1.39 | | | | 2.43 | |  |
|  | [95% CI] | [2,32 ; 2.45] | | | | [2.19 ; 2.53] | | | | [1.32 ; 1.46] | | | | [2.34 ; 2.52] | |  |
|  | SD | 1.21 | | | | 1.25 | | | | 1.20 | | | | 1.20 | |  |
|  | Min | 0 | | | | 0 | | | | 0 | | | | 0 | |  |
|  | Max | 6 | | | | 6 | | | | 6 | | | | 6 | |  |
| Perceived disability and/or emotional distress | Yes: n (%) | 689 | | (50.18%) | | 97 | | (44.91%) | | 592 | | (51.17%) | | 371 | (54.32%) |  |
|  | No: n (%) | 684 | | (49.82%) | | 119 | | (55.09%) | | 565 | | (48.83%) | | 312 | (45.68%) |  |
| Quality of life (seven-point Likert scale) | Mean | 4.57 | | | | 4.91 | | | | 4.51 | | | | 4.32 | |  |
|  | [95% CI] | [4.05 ; 4.63] | | | | [4.74 ; 5.09] | | | | [4.44 ; 4.58] | | | | [4.22 ; 4.41] | |  |
|  | SD | 1.27 | | | | 1.32 | | | | 1.25 | | | | 1.28 | |  |
|  | Min | 1 | | | | 1 | | | | 1 | | | | 1 | |  |
|  | Max | 7 | | | | 7 | | | | 7 | | | | 7 | |  |
| Health state satisfaction | Yes: n (%) | 874 | | (63.66%) | | 163 | | (75.46%) | | 711 | | (61.45%) | | 350 | (51.24%) |  |
|  | No: n (%) | 499 | | (36.35%) | | 53 | | (24.53%) | | 446 | | (38.55%) | | 333 | (48.74%) |  |
| **Decision-making characteristics** | | | | | | | | | | | | | | | |  |
| Most difficult decision | Take medication: n (%) | 278 | | (20.24%) | | 42 | | (19.44%) | | 236 | | (20.40%) | | 116 | (16.98%) |  |
|  | Get surgery: n (%) | 252 | | (18.35%) | | 45 | | (20.83%) | | 207 | | (17.89%) | | 125 | (18.30%) |  |
|  | Change my treatment: n (%) | 102 | | (7.43%) | | 15 | | (6.94%) | | 87 | | (7.52%) | | 28 | (6.94%) |  |
|  | Stop my treatment: n (%) | 60 | | (4.37%) | | 1 | | (0.46%) | | 59 | | (5.10%) | | 47 | (0.46%) |  |
|  | Change my lifestyle habits and behaviours: n (%) | 191 | | (13.91%) | | 34 | | (15.74%) | | 157 | | (13.57%) | | 80 | (11.71%) |  |
|  | Consult a rehabilitation professional: n (%) | 59 | | (4.30%) | | 3 | | (1.39%) | | 117 | | (4.84%) | | 35 | (5.12%) |  |
|  | Consult a complementary and alternative medicine professional: n (%) | 146 | | (10.63%) | | 29 | | (13.43%) | | 39 | | (10.11%) | | 73 | (10.83%) |  |
|  | Consult a mental-health professional: n (%) | 50 | | (3.63%) | | 11 | | (5.09%) | | 80 | | (3.37%) | | 23 | (3.37%) |  |
|  | Change the health care provider to manage my condition: n (%) | 93 | | (6.77%) | | 13 | | (6.02%) | | 116 | | (6.91%) | | 60 | (8.78%) |  |
|  | Undergo more diagnostic tests: n (%) | 139 | | (10.12%) | | 23 | | (10.65%) | | 3 | | (0.26%) | | 73 | (10.69%) |  |
|  | Other: n (%) | 3 | | (0.22%) | | 0 | | (0.00%) | | 0 | | (0.00%) | | 2 | (0.29%) |  |
| Prior knowledge on the available options | Yes, for all the options: n (%) | 337 | | (24.54%) | | 83 | | (38.43%) | | 254 | | (21.95%) | | 145 | (21.23%) |  |
|  | Yes, for certain options: n (%) | 771 | | (56.15%) | | 99 | | (45.83%) | | 672 | | (58.08%) | | 389 | (56.95%) |  |
|  | No: n (%) | 265 | | (19.30%) | | 34 | | (15.74%) | | 231 | | (19.97%) | | 149 | (21.81%) |  |
| Decision self-efficacy (11-point Likert scale) | Mean | 6.42 | | | | 7.84 | | | | 6.15 | | | | 5.72 | |  |
|  | [95% CI] | [6.30 ; 6.53] | | | | [7.55 ; 8.12] | | | | [6.03 ; 6.27] | | | | [5.56 ; 5.89] | |  |
|  | SD | 2.22 | | | | 2.13 | | | | 2.14 | | | | 2.18 | |  |
|  | Min | 0 | | | | 0 | | | | 0 | | | | 0 | |  |
|  | Max | 10 | | | | 10 | | | | 10 | | | | 10 | |  |
| Congruence between the chosen and preferred option | Yes: n (%) | 853 | | (65.51%) | | 172 | | (81.90%) | | 681 | | (58.86%) | | 327 | (51.17%) |  |
|  | No: n (%) | 198 | | (15.21%) | | 12 | | (5.71%) | | 186 | | (16.08%) | | 151 | (23.63%) |  |
|  | I don’t know, I let my health care provider decide for me: n (%) | 251 | | (19.28%) | | 26 | | (12.38%) | | 225 | | (19.45%) | | 161 | (25.20%) |  |
|  | Missing data: n (%) | 71 |  | (5.17%) | | 6 |  | (2.78%) | | 65 |  | (5.62%) | | 44 | (6.44%) |  |
| Health literacy (problem understanding what healthcare professionals said) | Always: n (%) | 29 | | (2.11%) | | 1 | | (0.46%) | | 28 | | (2.42%) | | 18 | (2.64%) |  |
|  | Often: n (%) | 156 | | (11.36%) | | 8 | | (3.70%) | | 148 | | (12.79%) | | 125 | (18.30%) |  |
|  | Sometimes: n (%) | 419 | | (30.52%) | | 44 | | (20.37%) | | 375 | | (32.41%) | | 255 | (37.34%) |  |
|  | Occasionally: n (%) | 372 | | (27.09%) | | 60 | | (27.78%) | | 312 | | (26.97%) | | 149 | (21.82%) |  |
|  | Never: n (%) | 397 | | (28.91%) | | 103 | | (47.69%) | | 294 | | (25.41%) | | 136 | (19.91%) |  |
| Perceived stress during the consultation | Yes: n (%) | 595 | | (43.36%) | | 69 | | (31.94%) | | 526 | | (45.46%) | | 350 | (51.24%) |  |
|  | No: n (%) | 685 | | 49.89%) | | 142 | | (65.74%) | | 543 | | (46.93%) | | 264 | (38.65%) |  |
|  | I don’t remember: n (%) | 93 | | (6.77%) | | 5 | | (2.31%) | | 88 | | (7.61%) | | 69 | (10.10%) |  |
| Involvement of important other(s) | Spouse: n (%) | 530 | | (38.60%) | | 77 | | (35.65%) | | 453 | | (39.15%) | | 257 | (37.63%) |  |
|  | Children: n (%) | 112 | | (8.16%) | | 13 | | (6.02%) | | 99 | | (8.56%) | | 67 | (9.81%) |  |
|  | Friends: n (%) | 169 | | (12.31%) | | 15 | | (6.94%) | | 154 | | (13.31%) | | 97 | (14.20%) |  |
|  | Professional occupation in religion: n (%) | 45 | | (3.28%) | | 1 | | (0.46%) | | 44 | | (3.80%) | | 34 | (4.98%) |  |
|  | Other member of close family: n (%) | 192 | | (13.98%) | | 30 | | (13.89%) | | 162 | | (14.00%) | | 90 | (13.18%) |  |
|  | Other: n (%) | 5 | | (0.36%) | | 1 | | (0.46%) | | 4 | | (0.35%) | | 3 | (0.44%) |  |
|  | Nobody: n (%) | 595 | | (43.34%) | | 112 | | (51.85%) | | 483 | | (41.75%) | | 282 | (41.29%) |  |
| Perception of assumed decision role | I made the decision alone: n (%) | 371 | | (27.02%) | | 61 | | (28.24%) | | 310 | | (26.79%) | | 198 | (28.99%) |  |
|  | I made the decision alone but considered the opinion of my health care providers: n (%) | 436 | | (31.76%) | | 57 | | (26.39%) | | 379 | | (32.76%) | | 213 | (31.19%) |  |
|  | My health care providers and I decided together, equally: n (%) | 372 | | (27.09%) | | 78 | | (36.11%) | | 294 | | (25.41%) | | 143 | (20.94%) |  |
|  | My health care providers made the decision but considered my opinion: n (%) | 126 | | (9.18%) | | 18 | | (8.33%) | | 108 | | (9.33%) | | 73 | (10.69%) |  |
|  | My health care providers made the decision alone: n (%) | 68 | | (4.95%) | | 2 | | (0.93%) | | 66 | | (5.70%) | | 56 | (8.20%) |  |
| Congruence between assumed and preferred decision role | No: n (%) | 688 | | (50.11%) | | 71 | | (32.87%) | | 617 | | (53.33%) | | 415 | (60.76%) |  |
|  | Yes: n (%) | 685 | | (49.89%) | | 145 | | (67.13%) | | 540 | | (46.67%) | | 268 | (39.24%) |  |
| Considered elements during the decision-making process | Option’s cost: n (%) | 369 | | (26.87%) | | 52 | | (24.07%) | | 317 | | (27.40%) | | 182 | (26.64%) |  |
|  | Pressure from others: n (%) | 120 | | (8.74%) | | 12 | | (5.56%) | | 108 | | (9.33%) | | 76 | (11.13%) |  |
|  | Time to implement the option: n (%) | 256 | | (18.64%) | | 40 | | (18.52%) | | 216 | | (18.67%) | | 127 | (18.59%) |  |
|  | Time before potential outcome: n (%) | 324 | | (23.60%) | | 57 | | (26.34%) | | 267 | | (23.08%) | | 158 | (23.13%) |  |
|  | Delay in accessing the option: n (%) | 234 | | (17.04%) | | 35 | | (16.20%) | | 199 | | (17.20%) | | 125 | (18.30%) |  |
|  | Potential benefits: n (%) | 678 | | (49.38%) | | 134 | | (62.04%) | | 544 | | (47.02%) | | 291 | (42.61%) |  |
|  | Potential harms: n (%) | 581 | | (42.31%) | | 87 | | (40.28%) | | 494 | | (42.70%) | | 266 | (38.94%) |  |
|  | Option’s consequences on your social, familial, or affective life: n (%) | 359 | | (26.15%) | | 62 | | (28.70%) | | 297 | | (25.67%) | | 176 | (25.77%) |  |
|  | Option’s consequences on your diet and consumptions: n (%) | 232 | | (16.90%) | | 37 | | (17.13%) | | 195 | | (16.85%) | | 108 | (15.81%) |  |
|  | Option’s consequences on a potential pregnancy: n (%) | 54 | | (3.93%) | | 8 | | (3.70%) | | 46 | | (3.98%) | | 37 | (5.42%) |  |
|  | Option’s consequences on your leisure: n (%) | 329 | | (23.96%) | | 62 | | (28.70%) | | 267 | | (23.08%) | | 150 | (21.96%) |  |
|  | Option’s consequences on your work or occupation: n (%) | 342 | | (24.91%) | | 57 | | (26.39%) | | 285 | | (24.63%) | | 161 | (23.57%) |  |
|  | Option’s consequences on your mobility: n (%) | 412 | | (30.01%) | | 73 | | (33.80%) | | 339 | | (29.30%) | | 187 | (27.38%) |  |
|  | Environmental impacts of the option: n (%) | 60 | | (4.37%) | | 4 | | (1.85%) | | 56 | | (4.84%) | | 33 | (4.83%) |  |
|  | Other: n (%) | 12 | | (0.87%) | | 3 | | (1.39%) | | 9 | | (0.78%) | | 5 | (0.73%) |  |
|  | No element: n (%) | 85 | | (6.19%) | | 18 | | (8.33%) | | 64 | | (5.53%) | | 48 | (7.03%) |  |
| Decisional conflict (0-100) | Mean | 30.76 | | | | 14.92 | | | | 33.72 | | | | 38.92 | |  |
|  | [95% CI] | [29.86 ; 31.66] | | | | [13.05 ; 16.79] | | | | [32.80 ; 34.63] | | | | [37.77 ; 40.08] | |  |
|  | SD | 17.02 | | | | 13.96 | | | | 15.87 | | | | 15.4 | |  |
|  | Min | 0 | | | | 0 | | | | 0 | | | | 0 | |  |
|  | Max | 93.75 | | | | 81.2 | | | | 93.75 | | | | 93.8 | |  |
| **Impacts of the chosen option** | | | | | | | | | | | | | | | |  |
| Impact of the chosen option on the treatment burden | Less workload: n (%) | 223 | | (16.24%) | | 36 | | (16.67%) | | 187 | | (16.16%) | | 106 | (15.20%) |  |
|  | No supplementary workload: n (%) | 515 | | (37.51%) | | 96 | | (44.44%) | | 419 | | (36.21%) | | 228 | (33.38%) |  |
|  | Low overload: n (%) | 360 | | (26.22%) | | 50 | | (23.15%) | | 310 | | (26.79%) | | 184 | (26.94%) |  |
|  | Moderate overload: n (%) | 218 | | (15.88%) | | 29 | | (13.43%) | | 189 | | (16.34%) | | 124 | (18.15%) |  |
|  | High overload: n (%) | 57 | | (4.15%) | | 5 | | (2.31%) | | 52 | | (4.49%) | | 41 | (6.00%) |  |
| Impact of the chosen option on the family (family burden) | Daily activity: n (%) | 122 | | (8.88%) | | 12 | | (5.56%) | | 110 | | (9.51%) | | 81 | (11.86%) |  |
|  | Household: n (%) | 249 | | (18.13%) | | 31 | | (14.35%) | | 218 | | (18.84%) | | 140 | (20.50%) |  |
|  | My family had to spend more time listening to my concerns: n (%) | 202 | | (14.71%) | | 23 | | (10.65%) | | 179 | | (15.47%) | | 114 | (16.69%) |  |
|  | Family interaction, routine, leisure and social network: n (%) | 130 | | (9.47%) | | 7 | | (3.24%) | | 123 | | (10.63%) | | 94 | (13.76%) |  |
|  | Economic implications: n (%) | 132 | | (9.61%) | | 10 | | (4.63%) | | 122 | | (10.54%) | | 97 | (14.20%) |  |
|  | Positive influence: n (%) | 239 | | (17.41%) | | 68 | | (31.48%) | | 171 | | (14.78%) | | 74 | (10.83%) |  |
|  | Other: n (%) | 5 | | (0.36%) | | 0 | | (0.00%) | | 5 | | (0.43%) | | 5 | (0.73%) |  |
