# Supplementary material for: People Living with Chronic Pain Experience a High Prevalence of Decision Regret in Canada: A Pan-Canadian Online Survey
Source: Med Decis Making. 2025 Mar 22;45(4):462–79. doi: 10.1177/0272989X251326069 (PMC11992647; doi:10.1177/0272989X251326069)
Supplement: sj-docx-4-mdm-10.1177_0272989X251326069 – Supplemental material for People Living with Chronic Pain Experience a High Prevalence of Decision Regret in Canada: A Pan-Canadian Online Survey [file sj-docx-4-mdm-10.1177_0272989X251326069.docx]

**Supplementary Material 4:**

1. Distribution of the Decision Regret Scale scores


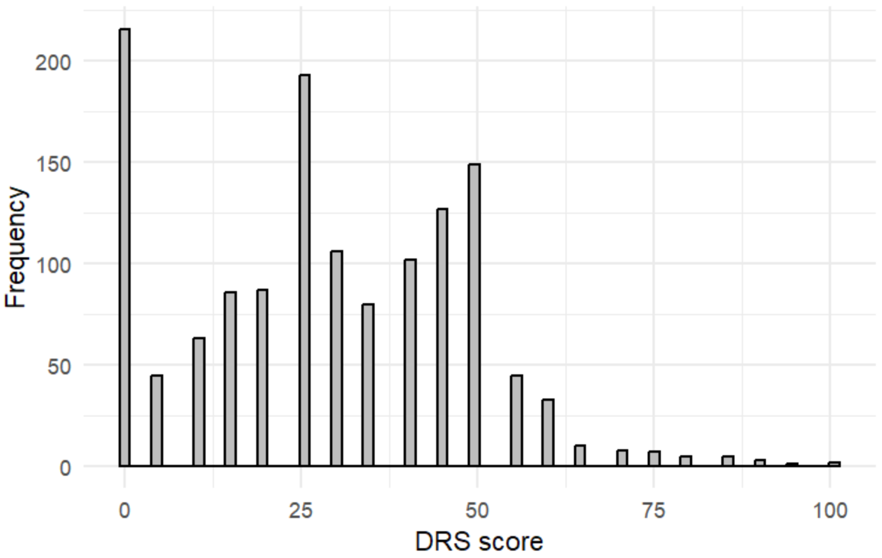


1. Descriptive results of the Decision Regret Scale score for each difficult decision.

| **Difficult decision** | **Mean [95%CI]** | **SD** | **Median** | **Q1** | **Q3** |
| --- | --- | --- | --- | --- | --- |
| Should I take medication or not? | 26.85 [24.6 ; 29.1] | 18.7 | 25 | 11.2 | 45 |
| Should I get surgery or not? | 27.2 [24.7 ; 29.7] | 19.9 | 25 | 10 | 45 |
| Should I change my treatment? | 30.59 [26.5 ; 34.7] | 20.9 | 25 | 20 | 45 |
| Should I stop my treatment? | 41.17 [36.7 ; 45.6] | 17.3 | 45 | 30 | 50 |
| Should I change my lifestyle habits and behaviours? | 26.1 [23.3 ; 28.9] | 19.7 | 25 | 10 | 40 |
| Should I consult a rehabilitation professional? | 34.83 [30.3 ; 39.3] | 17.3 | 40 | 25 | 50 |
| Should I consult a complementary and alternative medicine professional (e.g., chiropractor, osteopath, naturopath, acupuncture) | 27.6 [24.4 ; 30.8] | 19.5 | 30 | 10 | 43.8 |
| Should I consult a mental-health professional? | 25.4 [20.0 ; 30.8] | 18.9 | 25 | 7.5 | 38.8 |
| Should I change the health care provider to manage my condition? | 33.44 [29.5 ; 37.4] | 19.4 | 35 | 20 | 50 |
| Should I undergo more diagnostic tests? | 28.88 [25.6 ; 32.1] | 19.5 | 30 | 15 | 45 |
| Other(s), please specify | 46.67 [-15.8 ; 109.0] | 25.2 | 25 | 35 | 60 |

***Q1:*** First quartile, ***Q3:*** Third quartile, ***SD:*** Standard deviation, ***95%CI:*** 95% confidence interval
