# Supplementary material for: People Living with Chronic Pain Experience a High Prevalence of Decision Regret in Canada: A Pan-Canadian Online Survey
Source: Med Decis Making. 2025 Mar 22;45(4):462–79. doi: 10.1177/0272989X251326069 (PMC11992647; doi:10.1177/0272989X251326069)
Supplement: sj-docx-5-mdm-10.1177_0272989X251326069 – Supplemental material for People Living with Chronic Pain Experience a High Prevalence of Decision Regret in Canada: A Pan-Canadian Online Survey [file sj-docx-5-mdm-10.1177_0272989X251326069.docx]

**Supplementary Material 5:** Results of the complete case analysis.

| **Personal characteristics** |  |  |  |  |
| --- | --- | --- | --- | --- |
| Variable | Beta (imputed) | 95% CI (imputed) | Beta  (CCA) | 95% CI  (CCA) |
| Age (per SD unit) | -1.4* | [-2.5 ; -0.2] | -1.0 | [-2.3 ; 0.3] |
| Cultural and ethnical backgrounds |  |  |  |  |
| North American | REF | REF | REF | REF |
| European | 0.2 | [-1.8 ; 2.1] | -0.1 | [-2.2 ; 2.0] |
| Asian  Aboriginal  Other  Prefer not to say | 3.2  3.3  4.3  7.1* | [-0.2 ; 6.6]  [-0.4 ; 7.0]  [-0.3 ; 8.8]  [2.1 ; 12.0] | 2.7  1.1  4.6  -4.4 | [-1.3 ; 6.8]  [-3.1 ; 5.2]  [-0.9 ; 10.0]  [-17.1 ; 8.2] |
| Education level |  |  |  |  |
| Above the bachelor’s level | REF | REF | REF | REF |
| Bachelor’s degree | -0.9 | [-3.6 ; 1.8] | -0.6 | [-3.6 ; 2.4] |
| University diploma below bachelor’s level | 2.4 | [-1.0 ; 5.7] | 2.9 | [-0.9 ; 6.7] |
| College, CEGEP or other non-university diploma | 1.8 | [-0.9 ; 4.5] | 1.5 | [-1.6 ; 4.6] |
| High school or equivalency | 0.9 | [-2.0 ; 3.9] | -0.3 | [-3.7 ; 3.0] |
| Less than high school or equivalency | 10.3* | [4.3 ; 16.3] | 8.9* | [1.7 ; 16.1] |
| Geographical area |  |  |  |  |
| Urban | REF | REF | REF | REF |
| Rural | 0.07 | [-2.5 ; 2.6] | 0.9 | [-2.0 ; 3.9] |
| Household income (in CAD) |  |  |  |  |
| Less than 50,000 | REF | REF | REF | REF |
| 50,000 to less than 60,000 | 1.6 | [-1.1 ; 4.3] | 1.2 | [-1.8 ; 4.3] |
| 60,000 to less than 80,000 | 2.1 | [-0.6 ; 4.8] | 1.5 | [-1.6 ; 4.5] |
| 80,000 to less than 100,000 | 2.0 | [-0.8 ; 4.7] | 2.1 | [-1.0 ; 5.2] |
| 100,000 or more | 0.7 | [-1.8 ; 3.2] | 1.1 | [-1.7 ; 4.0] |
| Pain duration (per SD unit) | -0.5 | [-1.4 ; 0.4] | -0.8 | [-1.8 ; 0.1] |
| Quality of life | -0.2 | [-0.9 ; 0.6] | 0.2 | [-0.8 ; 1.1] |
| Sex |  |  |  |  |
| Male | REF | REF | REF | REF |
| Female | -1.6 | [-3.3 ; 0.2] | -3.3* | [-5.3 ; -1.4] |
| Intersex | 0.8 | [-14.1 ; 15.7] | -3.9 | [-24.9 ; 17.1] |
| Religious or spiritual affiliation |  |  |  |  |
| No religious or spiritual affiliation | REF | REF | REF | REF |
| Christian | -0.9 | [-3.7 ; 2.0] | -1.4 | [-4.7 ; 1.9] |
| Other religion or spiritual affiliation | -1.4 | [-3.2 ; 0.4] | -2.0 | [-4.0 ; 1.9] |
| Perceived disability and/or emotional distress |  |  |  |  |
| Yes | REF | REF | REF | REF |
| No | -0.4 | [-2.1 ; 1.3] | -1.0 | [-3.1 ; 1.0] |
| Health state satisfaction |  |  |  |  |
| Yes | REF | REF | REF | REF |
| No | 4.0* | [2.1 ; 5.9] | 4.1* | [1.9 ; 6.3] |
| Comorbidity |  |  |  |  |
| No comorbidity | -1.8 | [-4.4 ; 0.7] | -1.8 | [-4.7 ; 1.1] |
| Diabetes | -0.8 | [-3.2 ; 1.6] | -0.7 | [-3.4 ; 2.1] |
| Mental-health disorders | -2.5 | [-4.7 ; 0.4] | -1.6 | [-4.0 ; 0.9] |
| Alcohol-related disorders | 2.8 | [-1.7 ; 7.2] | 2.3 | [-2.7 ; 7.3] |
| Substance-related disorders | 0.5 | [-3.8 ; 4.7] | 2.8 | [-2.0 ; 7.5] |
| Sleep disorders | -0.1 | [-2.0 ; 1.8] | -0.4 | [-2.6 ; 1.8] |
| Hypertension | 0.8 | [-1.3 ; 2.9] | 1.2 | [-1.2 ; 3.6] |
| Disease of the respiratory system | 1.7 | [-0.7 ; 4.0] | 0.5 | [-2.2 ; 3.2] |
| Other | 0.7 | [-1.9 ; 3.4] | 0.6 | [-2.3 ; 3.5] |
| Marital status |  |  |  |  |
| Married / living common law | REF | REF | REF | REF |
| Never married | -0.9 | [-3.2 ; 1.5] | -0.2 | [-2.8 ; 2.5] |
| Separated / divorced / widowed | -0.1 | [-2.6 ; 2.4] | 0.6 | [-2.3 ; 3.5] |
| Number of people in the household | 0.4 | [-0.4 ; 1.1] | 0.5 | [-0.3 ; 1.4] |
| First learned language |  |  |  |  |
| English | REF | REF | REF | REF |
| French | -1.5 | [-3.8 ; 0.8] | -2.1 | [-4.5 ; 0.2] |
| An Aboriginal language | 24.0* | [3.4 ; 44.7] | 51.2* | [22.3 ; 80.1] |
| Other | 0.8 | [-2.3 ; 3.8] | -0.8 | [-4.4 ; 2.8] |
| Pain location |  |  |  |  |
| Head or face | -0.2 | [-2.4 ; 2.1] | -0.1 | [-2.7 ; 2.6] |
| Abdominal | -1.6 | [-4.4 ; 1.3] | -1.4 | [-4.7 ; 1.9] |
| Chest | 2.5 | [-1.2 ; 6.2] | 4.0 | [-0,2 ; 8.3] |
| Limbs (upper or lower) | -1.0 | [-2,7 ; 0,8] | -2.0 | [-4.0 ; 0.1] |
| Back | -1.3 | [-3.2 ; 0.6] | -1.5 | [-3.6 ; 0.7] |
| Pelvic | 0.5 | [-2.1 ; 3.0] | 2.0 | [-0.9 ; 4.8] |
| **Decision-making characteristics** | | |  |  |
| Variable | Beta  (imputed) | 95% CI  (imputed) | Beta  (CCA) | 95% CI  (CCA) |
| Most difficult decision |  |  |  |  |
| Take medication | REF | REF | REF | REF |
| Get surgery | 0.1 | [-2.6 ; 2.8] | 1.2 | [-1.9 ; 4.2] |
| Change treatment | 1.1 | [-2.3 ; 4.5] | 1.3 | [-2.9 ; 5.4] |
| Stop my treatment | 8.0* | [3.7 ; 12.1] | 10.4* | [5.4 ; 15.4] |
| Change lifestyle habits and behaviors | -1.6 | [-4.5 ; 1.3] | -1.2 | [-4.5 ; 2.1] |
| Consult a rehabilitation professional | 1.9 | [-2.3 ; 6.1] | 3.7 | [-1.3 ; 8.7] |
| Consult a CAM professional | -0.1 | [-3.2 ; 3.0] | -0.1 | [-3.5 ; 3.4] |
| Consult a mental-health professional | -3.9 | [-8.4 ; 0.7] | -2.3 | [-8.2 ; 3.6] |
| Change the healthcare provider | -1.0 | [-4.6 ; 2.7] | -0.8 | [-5.0 ; 3.5] |
| Undergo more diagnostic tests | -1.5 | [-4.6 ; 1.6] | -0.5 | [-4.2 ; 3.1] |
| Other | 10.8 | [-6.2 ; 27.8] | 6.6 | [-14.2 ; 27.4] |
| Prior knowledge on the options |  |  |  |  |
| On all the options | REF | REF | REF | REF |
| On certain options | -1.4 | [-3.4 ; 0.6] | -0.9 | [-3.2 ; 1.4] |
| No prior knowledge | -3.2* | [-5.9 ; -0.5] | -2.7 | [-5.8 ; 0.5] |
| Decision self-efficacy | -0.4 | [-0.9 ; 0.1] | -0.4 | [-1.0 ; 0.1] |
| Health literacy (problem understanding what doctor says) |  |  |  |  |
| Never | REF | REF | REF | REF |
| Occasionally | 1.3 | [-0.8 ; 3.4] | 1.5 | [-0.9 ; 3.9] |
| Sometimes | 1.9 | [-0.3 ; 4.1] | 0.9 | [-1.6 ; 3.5] |
| Often | 6.0* | [3.0 ; 9.0] | 5.4* | [1.9 ; 8.9] |
| Always | 4.3 | [-1.7 ; 10.3] | 3.6 | [-3.0 ; 10.2] |
| Congruence between chosen and preferred option |  |  |  |  |
| Yes | REF | REF | REF | REF |
| No | 3.4* | [0.8 ; 6.0] | 3.6* | [0.8 ; 6.4] |
| I don’t know, I let my health care professional decide for me | 2.0 | [-0.2 ; 4.3] | 1.3 | [-1,3 ; 3.9] |
| Perceived stress during the consultation |  |  |  |  |
| No | REF | REF | REF | REF |
| Yes | -1.1 | [-2.9 ; 0.8] | -0.7 | [-2.8 ; 1.4] |
| Decisional conflict (per SD unit) | 8.9* | [7.7 ; 10.0] | 8.5* | [7.2 ; 9.8] |
| Involvement of important other(s) |  |  |  |  |
| Nobody | -1.2 | [-3.2 ; 0.8] | -1.6 | [-3.8 ; 0.7] |
| Family | -0.6 | [-3.2 ; 2.0] | -0.3 | [-3.2 ; 2.6] |
| Friend | 2.8 | [0.2 ; 5.4] | 2.3 | [-0.6 ; 5.2] |
| Professional occupation in religion | 2.5 | [-2.1 ; 7.2] | 2.9 | [-2.2 ; 8.0] |
| Other | -0.6 | [-13.7 ; 12.4] | -0.4 | [-16.9 ; 16.1] |
| Perception of assumed decision role |  |  |  |  |
| I made the decision alone | REF | REF | REF | REF |
| I made the decision alone but considered the opinion of my health care provider | -0.4 | [-2.5 ; 1.7] | -0.9 | [-3.3 ; 1.6] |
| My health care providers and I decided together, equally | 0.4 | [-1.9 ; 2.6] | -0.5 | [-3.1 ; 2.2] |
| My health care providers made the decision but considered my opinion | -0.97 | [-4.1 ; 2.1] | -2.4 | [-6.0 ; 1.2] |
| My health care providers made the decision alone | 5.3* | [1.4 ; 9.3] | 4.3 | [-0.2 ; 8.8] |
| Congruence between assumed and preferred role decision |  |  |  |  |
| No | REF | REF | REF | REF |
| Yes | -2.2* | [-3.9 ; -0.6] | -2.2* | [-4.2 ; -0.3] |
| Considered elements during the decision-making process |  |  |  |  |
| Option’s cost | -0.4 | [-2.2 ; 1.5] | -0.2 | [-2.3 ; 1.9] |
| Pressure from others | 1.3 | [-1.6 ; 4.2] | 1.6 | [-1.8 ; 5.0] |
| Time to implement the option | -0.4 | [-2.7 ; 1.8] | -0.5 | [-3.0 ; 2.1] |
| Time before potential outcome | 0.2 | [-1.8 ; 2.2] | -1.7 | [-4.0 ; 0.7] |
| Delay in accessing the option | 0.1 | [-2.2 ; 2.4] | 0.6 | [-2.0 ; 3.2] |
| Potential benefits | -1.9* | [-3.6 ; -0.1] | -1.5 | [-3.4 ; 0.5] |
| Potential harms | -0.1 | [-1.9 ; 1.7] | 0.2 | [-1.9 ; 2.3] |
| Option’s consequences on your social, familial, or affective life | -1.1 | [-3.1 ; 0.9] | -1.0 | [-3.3 ; 1.2] |
| Option’s consequences on your diet and consumptions | -1.4 | [-3.7 ; 0.8] | -0.6 | [-3.1 ; 1.9] |
| Option’s consequences on a potential pregnancy | 2.7 | [-1.5 ; 7.0] | 2.3 | [-2.4 ; 7.1] |
| Option’s consequences on your leisure | 0.4 | [-1.8 ; 2.4] | 0.3 | [-2.0 ; 2.6] |
| Option’s consequences on your work or occupation | 0.4 | [-1.7 ; 2.4] | -0.1 | [-2.4 ; 2.2] |
| Option’s consequences on your mobility | -0.6 | [-2.5 ; 1.4] | -0.8 | [-3.0 ; 1.5] |
| Environmental impacts of the option | -0.4 | [-4.5 ; 3.6] | 0.2 | [-4.5 ; 4.8] |
| Other | -1.9 | [-10.6 ; 6.8] | -11.3* | [-22.0 ; -0.7] |
| **Influences of the chosen option** | | |  |  |
| Variable | Beta  (imputed) | 95% CI  (imputed) | Beta  (CCA) | 95% CI  (CCA) |
| Influence of the chosen option on the treatment burden |  |  |  |  |
| No supplementary workload | REF | REF | REF | REF |
| Less workload | -0.8 | [-3.2 ; 1.6] | -2.2 | [-4.9 ; 0.5] |
| Low overload | -0.6 | [-2.6 ; 1.4] | -1.1 | [-3.4 ; 1.2] |
| Moderate overload | -0.4 | [-2.9 ; 2.0] | -1.4 | [-4.3 ; 1.4] |
| High overload | 3.6 | [-0.8 ; 7.9] | 2.7 | [-2.2 ; 7.6] |
| Influence of the chosen option on the family |  |  |  |  |
| Influence on daily activities | 1.7 | [-1.2 ; 4.6] | 1.2 | [-2.1 ; 4.5] |
| Influence on household | -0.60 | [-2.8 ; 1.6] | -0.9 | [-3.3 ; 1.5] |
| My family had to spend more time listening to my concerns | -0.20 | [-2.6 ; 2.2] | -0.9 | [-3.5 ; 1.8] |
| Negative impacts on the family interaction | 4.2* | [1.3 ; 7.0] | 5.8* | [2.6 ; 9.0] |
| Negative economic implications | 4.2* | [1.3 ; 7.0] | 5.1* | [1.9 ; 8.3] |
| Positive influence | -5.3* | [-7.4 ; -3.1] | -5.0* | [-7.3 ; -2.6] |
| Other | 12.2 | [-0.8 ; 25.1] | 14.8* | [0.5 ; 29.1] |
| **Adjustment variable** | | |  |  |
| Variable | Beta  (imputed) | 95% CI  (imputed) | Beta  (CCA) | 95% CI  (CCA) |
| Accuracy of the recall of the consultation | 0.1 | [-0.1 ; 0.1] | 0.1 | [-0.1 ; 0.1] |

***CCA***: Complete case analysis, ***95%CI***: 95% Confidence Interval
